# AcconPred: Predicting Solvent Accessibility and Contact Number Simultaneously by a Multitask Learning Framework under the Conditional Neural Fields Model

**DOI:** 10.1155/2015/678764

**Published:** 2015-08-03

**Authors:** Jianzhu Ma, Sheng Wang

**Affiliations:** ^1^Toyota Technological Institute at Chicago, 6045 S. Kenwood Avenue, Chicago, IL 60637, USA; ^2^Department of Human Genetics, University of Chicago, E. 58th Street, Chicago, IL 60637, USA

## Abstract

*Motivation*. The solvent accessibility of protein residues is one of the driving forces of protein folding, while the contact number of protein residues limits the possibilities of protein conformations. The de novo prediction of these properties from protein sequence is important for the study of protein structure and function. Although these two properties are certainly related with each other, it is challenging to exploit this dependency for the prediction. *Method*. We present a method AcconPred for predicting solvent accessibility and contact number simultaneously, which is based on a shared weight multitask learning framework under the CNF (conditional neural fields) model. The multitask learning framework on a collection of related tasks provides more accurate prediction than the framework trained only on a single task. The CNF method not only models the complex relationship between the input features and the predicted labels, but also exploits the interdependency among adjacent labels. *Results*. Trained on 5729 monomeric soluble globular protein datasets, AcconPred could reach 0.68 three-state accuracy for solvent accessibility and 0.75 correlation for contact number. Tested on the 105 CASP11 domain datasets for solvent accessibility, AcconPred could reach 0.64 accuracy, which outperforms existing methods.

## 1. Introduction

The solvent accessibility of a protein residue is the surface area of the residue that is accessible to a solvent, which was first described by Lee and Richards [1] in 1971. During the process of protein folding, the residue solvent accessibility plays a very important role as it is related to the spatial arrangement and packing of the protein [2], which is depicted as the hydrophobic effect [3]. Specifically, the trends of the hydrophobic residues to be buried in the interior of the protein and the hydrophilic residues to be exposed to the solvent form the hydrophobic effect that functions as the driving force for the folding of monomeric soluble globular proteins [4–6].

Solvent accessibility can help protein structure prediction in two aspects. (1) Since solvent accessibility is calculated on all-atom protein structure coordinates, it encodes the global information of the 3D protein structure into a 1D feature, which makes solvent accessibility as an excellent piece of complementary information to the other local 1D features such as secondary structure [7–9], structural alphabet [10–12], or backbone torsion angles [13–15]. (2) Compared to the other global information such as the contact map [16, 17] or the distance map [18, 19], solvent accessibility shares similar property of the other local 1D feature that it could be predicted into a relatively accurate level [20]. Therefore, the predicted solvent accessibility has been widely utilized for detection as well as threading of remote homologous proteins [21–23] and quality assessment of protein models [24, 25].

The contact number is yet another kind of 1D feature that encodes the 3D information, which is related to, but different from, solvent accessibility [26]. The contact number of a protein residue is actually the result of protein folding. It has been suggested that, given the contact number for each residue, the possibilities of protein conformations that satisfy the contact number constraints are very limited [27]. Thus, the predicted contact numbers of a protein may serve as useful restraints for de novo structure prediction [26] or contact map prediction [28].

To predict the protein solvent accessibility, most methods first discretize it into two- or three-state labels based on the continuous relative solvent accessibility value [20]. Then these methods apply a variety of learning approaches for the prediction, such as neural networks [29–34], SVM (support vector machine) [35–37], Bayesian statistics [38], and nearest neighbor [20, 39]. Some other methods also attempt to directly predict the continuous absolute or relative solvent accessibility value [14, 34, 40–42].

Comparing with the solvent accessibility prediction, there are much fewer methods that deal with the prediction of contact number. For example, Kinjo et al. [26] employ linear regression analysis, Pollastri et al. [32] use neural networks, and Yuan [43] applies SVM.

Since a high dependency between the adjacent labels for both solvent accessibility and contact number exists [44], it is hard to utilize this information based on the previous proposed computational methods. For instance, neural network methods usually do not take the interdependency relationship among the labels of adjacent residues into consideration. Similarly, it is also challenging for SVM to deal with this dependency information [45]. Although hidden Markov model (HMM) [44] is capable of describing this dependency, it is challenging for HMM to model the complex nonlinear relationship between input protein features and the predicted solvent accessibility labels, especially when a large amount of heterogeneous protein features is available [45].

Recently, ACCpro5 [46] could reach almost perfect prediction of protein solvent accessibility by the aid of structural similarity in the protein template database. However, such approach might not perform well on those de novo folds or the sequences that cannot find any similar proteins in the database.

Although solvent accessibility and contact number are two different quantities, they are certainly related with each other, both reflecting the hydrophobic or hydrophilic atmosphere of each residue in the protein structure [26]. For example, a residue with a large contact number would probably be buried inside the core, whereas a residue with a small contact number would probably be exposed to the solvent. Therefore, a learning approach that could utilize this relationship to extract the universal representation of the features would be beneficial.

Here we present AcconPred (solvent accessibility and contact number prediction), available at http://ttic.uchicago.edu/~majianzhu/AcconPred_package_v1.00.tar.gz based on a shared weight multitask learning framework under the CNF (conditional neural fields) model. As a recently invented probabilistic graphical model, CNF [47] has been used for a variety of bioinformatics tasks [21–23, 45, 48–52]. Specifically, CNF is a perfect integration of CRF (Conditional Random Fields) [53] and neural networks. Besides modeling the nonlinear relationship between the input protein features and the predicted labels as what neural network does, CNF can also model the interdependency among adjacent labels as what CRF does.

It has been shown that a unified neural network architecture, trained simultaneously on a collection of related tasks, provides more accurate labelings than a network trained only on a single task [54]. A study by Caruana thus demonstrates the power of multitask learning that could extract the universal representation of the input features [55]. In AcconPred, we integrate multitask learning framework under the CNF model by sharing the weight of the neuron functions between the two tasks, followed by a stochastic gradient descent for training the parameters.

Last but not least, AcconPred can provide a probability distribution over all the possible labels. That is, instead of predicting a single label at each residue, AcconPred will generate the label probability distribution for solvent accessibility and contact number. Our testing data shows that AcconPred achieves better accuracy on solvent accessibility prediction and higher correlation on contact number prediction than the other methods.

## 2. Method

### 2.1. Preliminary Definition

#### 2.1.1. Calculating Solvent Accessibility from Native Protein Structure

We applied DSSP [7] to calculate the absolute accessible surface area for each residue in a protein. The relative solvent accessibility (RSA) of the residue X is calculated through dividing the absolute accessible surface area by the maximum solvent accessibility which uses Gly-X-Gly extended tripeptides [56]. In particular, these values are 210 (Phe), 175 (Ile), 170 (Leu), 155 (Val), 145 (Pro), 115 (Ala), 75 (Gly), 185 (Met), 135 (Cys), 255 (Trp), 230 (Tyr), 140 (Thr), 115 (Ser), 180 (Gln), 160 (Asn), 190 (Glu), 150 (Asp), 195 (His), 200 (Lys), and 225 (Arg), in units of Å^2^.

With the relative solvent accessibility value, the classification was divided into three states, say, buried (B), intermediate (I), and exposed (E), as in the literatures [14, 20]. In this work, the usage of 10% for B/I and 40% for I/E in the 3-state definition is based on the following two facts: (1) such division is close to the definition of previous method [20]; (2) at this cutoff, the background distribution for the three states in our training data is close to 1 : 1 : 1. A more comprehensive interpretation for this 10%/40% threshold is described in Results and shown in Figure 2.

#### 2.1.2. Calculating Contact Number from Native Protein Structure

To calculate the contact number for each residue, we followed similar definition from previous works [26, 43]. Basically, the contact number (CN) of the *i*th residue in a protein structure is the number of C-beta atoms from the other residues (excluding 5 nearest-neighbor residues) within the sphere of the radius 7.5 Å centered at the C-beta atom of the *i*th residue. We also limit the maximal contact number as 14 if the observed contact number is above 14, because such cases are rare in our training data. So for each residue, there are 15 states of contact number in total.

### 2.2. Datasets

#### 2.2.1. Training and Validation Data

Training and validation data were extracted from all monomeric, globular, and nonmembrane protein structures. They were downloaded from Protein Data Bank (PDB) [57] dated before May 1, 2014. The monomeric proteins were extracted according to the “Remark 350 Author Determined Biological Unit: Monomeric” recorded in the PDB file. To exclude those nonglobular proteins, we calculated the buried residue ratio (i.e., the percentage of the residues in buried state) for each protein and removed those proteins with <10% buried residue ratio. To exclude those membrane proteins, the PDBTM database [58] was employed.

The reason for using monomeric protein to predict solvent accessibility is based on the fact that the patterns in the surface of the monomeric proteins are different from those in the interface of the oligomeric proteins [59]. Again, the reason why we exclude the membrane proteins is that they have the opposite solvent accessibility pattern to those monomeric, globular soluble proteins. Furthermore, the 10% buried residue ratio cutoff was derived from statistics for the globular protein database [60].

Finally, we excluded proteins with length less than 50, having chain-breaks in the middle, and the 40% sequence identity was applied to remove redundancy. So in total we have 5729 monomeric, globular, and nonmembrane protein structures as our training and validation dataset (5-cross validation). The 5729 PDB IDs included in the training and validation datasets could be found in the Supplementary Material available online at http://dx.doi.org/10.1155/2015/678764.

#### 2.2.2. Testing Data

The testing data were collected from the CASP11 [61] targets containing 105 domains. Note that all CASP11 targets were released after May 1, 2014. The PDB structures for the 105 CASP11 testing datasets could be found in the Supplementary Files.

In order to compare with the existing programs, we further included the dataset from Yuan [43] as the testing data for contact number prediction. The 945 PDB IDs included in the Yuan dataset could be found in the Supplementary Files.

### 2.3. Protein Features

A variety of protein features have been studied by [14, 29–32, 41, 62, 63] to predict the solvent accessibility or the contact number. They could be categorized into three classes: evolution related, structure related, and amino acid related features, which will form our feature vector *F*(*i*) for residue *i*. Furthermore, since the solvent accessibility or the contact number for a certain residue could be influenced by its nearby residues in sequence, we then introduce a windows size *k* to capture this information. That is, we take the feature vectors from *F*(*i* − *k*), *F*(*i* − *k* + 1), …, *F*(*i*), …, *F*(*i* + *k* − 1), *F*(*i* + *k*) as the final input features for residue *i*. In this work we set the windows size *k* = 5.

#### 2.3.1. Evolution Related Features

Solvent accessibility as well as contact number of a residue has a strong relationship with the residue's substitution and evolution. Residues in the buried core and residues on the solvent-exposed surfaces were shown to have different substitution patterns due to different selection pressure [64]. Evolution information such as PSSM (position specific scoring matrix) and PSFM (position specific frequency matrix) generated by PSI-BLAST [65] has been used and proved to enhance the prediction performance. Here we use different evolution information from the HHM file generated by HHpred [66]. In particular, it first invokes PSI-BLAST with five iterations and *E*-value 0.001 and then computes the homology information for each residue combined with a context-specific background probability [67]. Overall, for each residue, we have 40 = 20 + 20 evolution related features.

#### 2.3.2. Structure Related Features

Local structural features are also very useful in predicting solvent accessibility, as indicated in [41]. Here we use the predicted secondary structure elements (SSEs) probability as the structure related features for each residue position. In particular, we use both 3-class and 8-class SSEs. The 3-class SSE is predicted by PSIPRED [8] which is more accurate but contains less information, while the 8-class secondary structure element is predicted by RaptorX-SS8 [45] which is less accurate but contains more information. Overall, for each residue, we have 11 = 8 + 3 structure related features.

#### 2.3.3. Amino Acid Related Features

Besides using position dependent evolutionary and structural features, we also use position independent features such as (a) physicochemical property, (b) specific propensity of being endpoints of an SS segment, and (c) correlated contact potential, for each amino acid. Specifically, physicochemical property has 7 values for each amino acid (shown in Table 1 from [68]); specific propensity of being endpoints of an SS segment has 11 values for each amino acid (shown in Table 1 from [69]); correlated contact potential has 40 values for each amino acid (shown in Table 3 from [70]). All these features have been studied in [45] for secondary structure elements prediction and in [21–23] for homology detection. Overall, for each residue, we have 58 = 7 + 11 + 40 amino acid dependent features.

### 2.4. Prediction Method

#### 2.4.1. CNF Model

**Figure 1:**
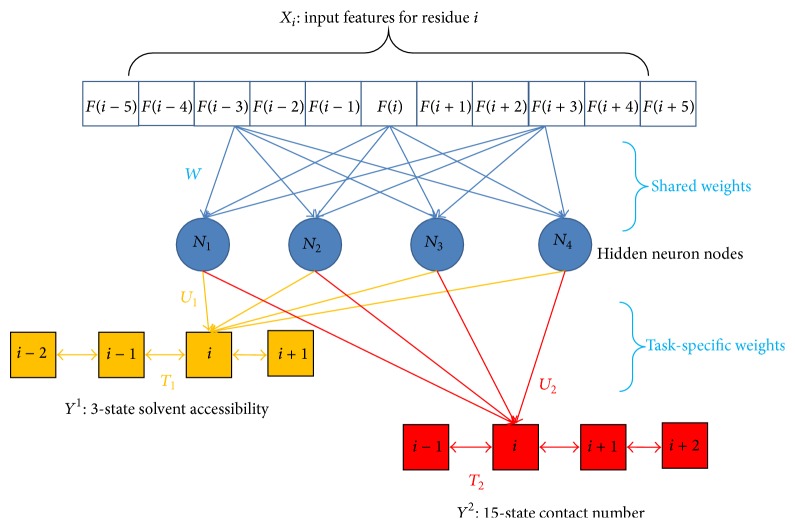
The shared weight multitask learning framework under the CNF (conditional neural fields) model for 3-state solvent accessibility and 15-state contact number prediction. CNF could model the relationship between input features *X* and label *Y* through a hidden layer of neuron nodes, which conduct nonlinear transformation of *X*. Note that the weight *W* from the input features to hidden neuron nodes is fixed for all tasks, while the weight *U* from neuron to label and the weight *T* from label to label are task-specific.

Conditional neural fields (CNF) [47] are probabilistic graphical models that have been extensively used in modeling sequential data [45, 49]. Given features on each residue on a protein sequence, we could compute the probability of each label for one residue and the transition probability for neighboring residues. Formally, for a given protein with length *L*, we denote its predicted labels (say, 3-state solvent accessibility or 15-state contact number) as **Y**( = (*Y*_1_,…, *Y*_*L*_)), where *Y*_*i*_ ∈ {1,2,…, *M*}, *M* = 3 for solvent accessibility prediction, and *M* = 15 for contact number prediction. We also represent the input features of a given protein by an *n* × *L* matrix **X**( = (*F*(1),…, *F*(*L*))), where *n* represents the number of hidden neurons and the *i*th column vector *F*(*i*) represents the protein feature vector associated with the *i*th residue, defined in the previous section. Then we can formulize the conditional probability of the predicted labels **Y** on protein feature matrix **X** as follows:

(1) PY ∣ X ∝exp⁡⁡+∑i=1L ∑j=1nϕYi,NjFi−k,…,Fi+k∑i=1L−1ψYi,Yi+1hhhhhhhh+∑i=1L ∑j=1nϕYi,NjFi−k,…,Fi+k,

where *ψ*(*Y*_*i*_, *Y*_*i*+1_) is the potential function defined on an edge connecting two nodes; *ϕ*(*Y*_*i*_, *N*_*j*_(*F*(*i* − *k*),…, *F*(*i* + *k*))) is the potential function defined at the position *i*; *N*_*j*_() is a hidden neuron function that does nonlinear transformation of input protein features; *k* is the window size. Formally, *ψ*() and *ϕ*() are defined as follows:

(2) $$\begin{array}{c} \psi \left(Y_{i},Y_{i+1}\right)=\underset{a,b}{\sum }t_{a,b}\delta \left(Y_{i}=a\right)\delta \left(Y_{i+1}=b\right),\\ \phi \left(Y_{i},N_{j}\right)=\sum_{a}u_{a,j}N_{j}\left(w_{j}^{T}f\left(i\right)\right)\delta \left(Y_{i}=a\right), \end{array}$$

where *δ*() is an indicator function; *f*(*i*) represents the final input features *F*(*i* − *k*),…, *F*(*i* + *k*) for residue *i*; *W*, *U*, and *T* are model parameters to be trained. Specifically, *W* is the parameter from the input features to hidden neuron nodes, *U* from neuron to label, and *T* from label to label, respectively; *a* and *b* represent predicted labels (see Figure 1). The details for the training and prediction of the CNF model could be found in [45]. One beneficial result of CNF is the probability output for each label at a position through a MAP (maximum a posteriori) procedure. These probabilities, generated by CNF models trained by different combinations of feature classes, could be further utilized as features for training a consensus CNF model.

#### 2.4.2. Multitask Learning Framework

Multitask learning (MTL) has recently attracted extensive research interest in the data mining and machine learning community [71–74]. It has been observed that learning multiple related tasks simultaneously often improves predicted accuracy [54]. Inspired by [75], a variety of functionally important protein properties, such as secondary structure and solvent accessibility, can be encoded as a labeling of amino acids and trained in multitask simultaneously under a deep neural network framework [75]. Here we propose a similar procedure for learning two tasks, say solvent accessibility and contact number, under a weight sharing CNF framework.

Specifically, assuming we have *T* related tasks, the “weight sharing” strategy implies that the parameters for the *N*_*j*_() function are shared between tasks. That is to say, the hidden neuron function that does nonlinear transformation of input protein features is shared for predicting solvent accessibility and contact number. The whole CNF framework includes the parameters *θ*_*t*_ = {*W*, *U*_*t*_, *T*_*t*_} for each task *t*. With this setup (i.e., only the neuron to label function *U* and the label to label function *T* are task-specific), the CNF framework automatically learns an embedding that generalizes across tasks in the first hidden neuron layers and learns features specific for the desired tasks in the second layers.

When using stochastic gradient descent to train the model parameters, we could carry out the following three steps: (a) select a task at random, (b) select a random training example for this task, and (c) compute the gradients of the CNF attributed to this task with respect to this example and update the parameters. Again, the probabilities generated by CNF models trained for different task could be utilized as features for training a consensus CNF model for a single task.

## 3. Results

We evaluate our program AcconPred on two prediction tasks, say solvent accessibility prediction and contact number prediction, on our own training data and CASP11 testing data. For contact number prediction, in order to compare with the existing programs, we further include the Yuan [43] dataset as the testing data. Besides using accuracy as the measurement for both solvent accessibility and contact number, we also use the following evaluation metrics for solvent accessibility, which includes precision (defined as TP/(TP + FN)), recall (defined as TP/(TP + FN)), and* F1* score (defined as 2TP/(2TP + FP + FN)), where TP, TN, FP, and FN are the numbers of the true positives, true negatives, false positives, and false negatives for a given dataset, respectively. To evaluate the performance of contact number, we also calculate the Pearson correlation between the predicted and the observed values.

In the following sections, we first give an interpretation of the 10%/40% threshold that defines the 3-state solvent accessibility. Then we evaluate the performance of AcconPred on the training data. Followed by briefly describing the programs to be compared, we show the outperformance of AcconPred with the existing programs on the testing data, which includes CASP11 and Yuan dataset.

### 3.1. Interpretation of the 10%/40% Threshold That Defines the 3-State Solvent Accessibility

Traditionally, predicting solvent accessibility using machine learning models is regarded as either 2, 3, or 10 labels of classification problem or a real value regression problem. There is no widely accepted criterion on how to classify the real value solvent accessibility into a finite number of discrete states such as buried, intermediate, and exposed. The reason is that, in a classification problem, with fewer labels we could get a more accurate prediction but at the same time lose lots of information by merging adjacent classes. This fact still holds between classification and regression because regression could be recognized as a kind of infinite labels prediction task with lower accuracy comparing with classification under the same situation.

Therefore, it is a tradeoff between using fewer labels of less information and using more labels less accurate. In addition, even for the same number of labels in the classification problem, the boundary for each label still needs to be finely determined. Remember that solvent accessibility represents the relative buried degree of one residue in the whole 3D protein so it is possible for two aligned residues on two structural related proteins to have different real value of accessibility in some range. To decide the range of each label is equal to giving a standard to judge if two residues with different solvent accessibilities can be aligned together.

**Figure 2:**
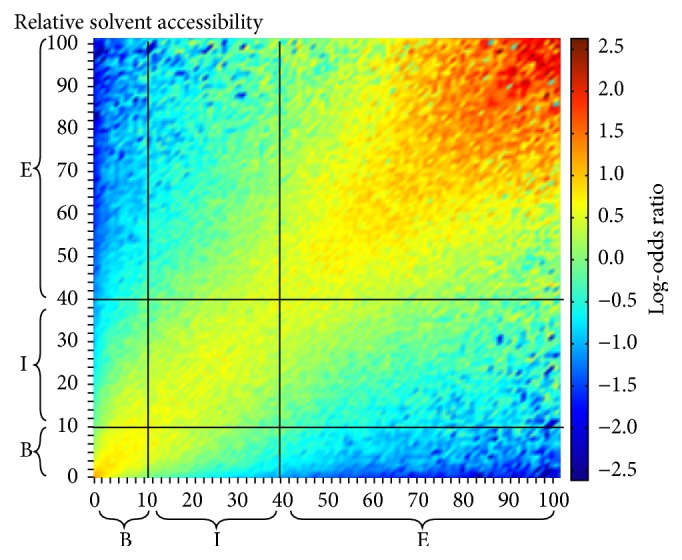
Log-odds ratio between the pair frequencies in the structure alignments and the background frequencies, with respect to the relative solvent accessibility in 1% unit. The thick black line indicates the boundaries at 10% and 40% to define the 3-label solvent accessibility, say buried (B), intermediate (I), and exposed (E).

In this work, the three discrete states on relative solvent accessibility with boundaries at 10% and 40% are used (see Figure 2). We could give an interpretation for such boundaries by all-against-all protein pairwise structure alignments [76–79] on our training data. Followed by filtering out the pairs with TM-score [80] lower than 0.65 which indicates that the two proteins have no obvious biological relevance [81], we calculate the log-odds ratio between the pair frequencies in the remaining structure alignments and the background frequencies, with respect to the relative solvent accessibility in 1% unit. As shown in Figure 2, the area with more red color means that the corresponding two relative solvent accessibilities on two aligned proteins have more chance to coappear in the structure alignments, while the area with more blue color is vice versa. As a result, it can be concluded that, under such boundaries, the within-class distance is low (with more yellow or red points), while the between-class distance is high (with more cyan or blue area).

### 3.2. Performance on Training Data

#### 3.2.1. Results for 3-State Solvent Accessibility Prediction

(*1) Precision, Recall, and F1 Score for Each Predicted Label*.  Table 1 gives detailed results for each label of solvent accessibility prediction, say buried, intermediate, and exposed. Besides the overall analysis in terms of precision, recall, and* F1* score, we also provide the subset analysis of the predicted label which is chosen according to the predicted probability. From this table, we observe that when predicted probability is above 0.8, both the predicted buried label and exposed label could reach about 0.9 accuracy. However, the prediction of the intermediate label is least accurate, which can be probably expected from the arbitrariness of the threshold between the three states [20].

(*2) Relative Importance of the Three Classes of Features*. As mentioned in the previous section, the features used in the training process consist of three classes: evolution related, structure related, and amino acid related, respectively. In order to estimate the impact of each class on 3-state solvent accessibility prediction, we apply each of them to train the model and perform the prediction.  Table 2 illustrates the prediction accuracy of different feature classes and different learning models, including single task learning model and multitask learning model. It could be observed that using amino acid related feature alone could reach 0.55 Q3 accuracy, and this accuracy could be largely increased by using the evolution related feature alone. It is interesting that although the structure related features are actually derived from the evolutionary information, the combination of all these three classes of features could reach 0.66 Q3 accuracy. Finally, we show that the performance improvement could be gained by performing multitask learning for 2% accuracy.

#### 3.2.2. Results for 15-State Contact Number Prediction

Table 3 illustrates the prediction accuracy of different feature classes and different learning models for 15-state contact number, with the same trend in Table 2 in 3-state solvent accessibility perdition. It should be noted that if the difference between the predicted contact number and the observed value is only 1 or 2, we still could tolerate the result.  Table 4 shows the prediction accuracy of different tolerance values, ranging from 0 to 3. If 1, 2, or 3 differences between the predicted contact number and the observed value are tolerated, the accuracy could reach 0.63, 0.83, and 0.93, respectively. The Pearson correlation score of AcconPred on the training data is 0.75.

### 3.3. Performance on Testing Data

#### 3.3.1. The Existing Programs to Be Compared

We compare AcconPred with three popular solvent accessibility prediction programs, say SPINE-X [14], SANN [20], and ACCpro5 [46], as well as two contact number prediction programs, say Kinjo's method [26] and Yuan's method [43]. For solvent accessibility prediction, SPINE-X is a neural network based method, whereas SANN is based on nearest neighbor. In contrast to these two methods that rely on protein sequence information alone, ACCpro5 exploits the additional structural information derived from PDB. For contact number prediction, both Kinjo's and Yuan's methods extract features from protein sequence information. However, Kinjo's method applies linear regression for the prediction, while Yuan's method employs SVM method.

#### 3.3.2. Results on CASP11 Data

Table 5 summarizes the results of three existing and well-known methods (say, SPINE-X, SANN, and ACCpro5, resp.) for predicting the 3-state solvent accessibility on the CASP11 105 domain cases. It should be noted that the original 3-state output of SPINE-X is based on the 25%/75% threshold, while SANN is 9%/36%. However, besides the discretized output, both SPINE-X and SANN also output predicted continuous relative solvent accessibility that ranges from 0 to 100%. So we use the same 10%/40% threshold as AcconPred to relabel the output from SPINE-X and SANN. Furthermore, the original output of ACCpro5 is 2-state which cut at 25%. Nonetheless, ACCpro5 also generates 20-state relative solvent accessibility at all thresholds between 0% and 95% at 5% increments. So in this case we could also easily transform the output of ACCpro5 into the 3-state at 10%/40% threshold. We observe that AcconPred could reach 0.65 Q3 accuracy, which is higher than SPINE-X, SANN, and ACCpro5 whose Q3 accuracies are 0.57, 0.61, and 0.58, respectively. All detailed results from SPINE-X, SANN, and ACCpro5 could be found in Supplementary Files.

We also calculate the Q15 prediction accuracy and correlation of AcconPred for 15-state contact number on CASP11 data. The results are 0.28 for Q15 and 0.71 for correlation, which is quite consistent with the results from the training data (0.3 for Q15 and 0.74 for correlation) and the Yuan data (0.28 for Q15 and 0.72 for correlation).

#### 3.3.3. Results on Yuan Data

Since the software of both Kinjo's method and Yuan's method is not available, we perform AcconPred on the training set from Yuan. It should be noted that the Yuan data (containing 945 PDB chains) were also the training data for Kinjo's method [26]. Because the same dataset is used for contact number prediction, we could directly extract the results of Kinjo's method and Yuan's method from their paper for the comparison analysis.  Table 6 summarizes the correlation results for Kinjo's method, Yuan's method, and AcconPred. We observe that our proposed method AcconPred outperforms the other methods significantly. The correlation score of AcconPred is 0.72, which is better than Kinjo's method (correlation score is 0.63) and Yuan's method (correlation score is 0.64).

## 4. Discussion and Future Work

In this work, we have presented AcconPred for predicting the 3-state solvent accessibility as well as the 15-state contact number for a given protein sequence. The method is based on a shared weight multitask learning framework under the CNF model. The overall performance of AcconPred for both solvent accessibility and contact number prediction is significantly better than the state-of-the-art methods.

There are two reasons why AcconPred could achieve this performance. (1) The CNF model not only captures the complex nonlinear relationship between the input protein features and the predicted labels, but also exploits interdependence among adjacent labels [45, 47]. (2) The shared weight multitask learning framework could incorporate the information of both solvent accessibility and contact number simultaneously during training [75].

Furthermore, the CNF model defines a probability distribution over the label space. The probability distribution, generated by CNF models trained on different combinations of feature classes (shown in Tables 2 and 3) for both solvent accessibility and contact number, could be further applied as the input feature to train a regression neural network model for predicting the continuous relative solvent accessibility. Meanwhile, the predicted contact number probability alone could be applied as topology constraints for the contact map prediction. It is suggested that the same framework of AcconPred could be applied to predict 10-state relative solvent accessibility, with 10% at each interval. Similar as in Table 4, we could also measure the prediction accuracy of different tolerance values for 10-state solvent accessibility.

Another uniqueness of our work is the training data, which excludes those “outlier” cases for solvent accessibility training, such as oligomer, membrane, and nonglobular proteins. This is because of the fact that these proteins have quite different solvent accessibility patterns with the monomeric soluble globular proteins. Recently, [82] pointed out that there were preferred chemical patterns of closely packed residues at the protein-protein interface. It implies that our training data that contains monomeric soluble globular proteins could serve as a control set for protein-protein interface prediction.

## Supplementary Material

Training/testing dataset as well as the CASP11 evaluation dataset could be found in the Supplementary Material. The CASP11 evaluation dataset contains all detailed results from SPINE-X, SANN, ACCpro5 and AcconPred.

## Figures and Tables

**Table 1:** Precision, recall, and *F*1 score for different evaluation dataset of 3-state solvent accessibility prediction.

Evaluation dataset	Precision	Recall	*F*1 score
^†^Buried overall	0.76	0.78	0.77
^‡^Buried >0.9	0.96	0.31	0.47
Buried >0.8	0.92	0.45	0.60
Buried >0.7	0.88	0.57	0.69
Buried >0.6	0.84	0.66	0.74
Buried >0.5	0.79	0.74	0.76
Buried >0.4	0.75	0.82	0.78

Intermediate overall	0.56	0.50	0.53
Intermediate >0.9	1.00	0.0001	0.002
Intermediate >0.8	0.82	0.006	0.01
Intermediate >0.7	0.74	0.06	0.11
Intermediate >0.6	0.67	0.19	0.30
Intermediate >0.5	0.61	0.38	0.47
Intermediate >0.4	0.55	0.61	0.58

Exposed overall	0.71	0.76	0.73
Exposed >0.9	0.94	0.11	0.20
Exposed >0.8	0.88	0.31	0.46
Exposed >0.7	0.83	0.47	0.60
Exposed >0.6	0.78	0.61	0.68
Exposed >0.5	0.74	0.72	0.73
Exposed >0.4	0.69	0.81	0.75

^†^Overall indicates the whole set of the predicted labels.

^‡^>0.9 indicates that the set of the predicted labels is chosen according to the predicted probability which is larger than 0.9.

**Table 2:** Prediction accuracy of different feature class and learning model for 3-state solvent accessibility.

Features	Evolution	Structure	Amino acid	^†^Combined single	^‡^Combined MTL

Q3 accuracy	0.64	0.59	0.55	0.66	**0.68**

^†^Combined single indicates that all classes of features, including evolution, structure, and amino acid, are used for training a single task model.

^‡^Combined MTL indicates that all classes of features are used for training a multitask learning model.

**Table 3:** Prediction accuracy of different feature class and learning models for 15-state contact number (with the same explanation as in Table 2).

Features	Evolution	Structure	Amino acid	Combined single	Combined MTL

Q15 accuracy	0.26	0.24	0.19	0.28	**0.30**

**Table 4:** Prediction accuracy of different tolerance values for 15-state contact number.

Tolerance	0	1	2	3

Accuracy	0.30	0.63	0.83	0.93

**Table 5:** Comparison results of the prediction accuracy of AcconPred with existing programs for 3-state solvent accessibility on the CASP11 dataset.

Method	SPINE-X	SANN	ACCpro5	AcconPred

Q3 accuracy	0.57	0.61	0.58	**0.64**

**Table 6:** Comparison results of the Pearson correlation score of AcconPred with existing programs for contact number prediction on the Yuan dataset.

Method	Kinjo	Yuan	AcconPred

Correlation	0.63	0.64	**0.72**

## References

[1] Lee B., Richards F. M. (1971). The interpretation of protein structures: estimation of static accessibility. *Journal of Molecular Biology*.

[2] Kauzmann W. (1959). Some factors in the interpretation of protein denaturation. *Advances in Protein Chemistry*.

[3] Dill K. A. (1990). Dominant forces in protein folding. *Biochemistry*.

[4] Chothia C. (1975). Structural invariants in protein folding. *Nature*.

[5] Rose G. D., Geselowitz A. R., Lesser G. J., Lee R. H., Zehfus M. H. (1985). Hydrophobicity of amino acid residues in globular proteins. *Science*.

[6] Sharp K. A. (1991). Extracting hydrophobic free energies from experimental data: relationship to protein folding and theoretical models. *Biochemistry*.

[7] Kabsch W., Sander C. (1983). Dictionary of protein secondary structure: pattern recognition of hydrogen-bonded and geometrical features. *Biopolymers—Peptide Science Section*.

[8] McGuffin L. J., Bryson K., Jones D. T. (2000). The PSIPRED protein structure prediction server. *Bioinformatics*.

[9] Frishman D., Argos P. (1995). Knowledge-based protein secondary structure assignment. *Proteins: Structure, Function, and Bioinformatics*.

[10] de Brevern A. G., Etchebest C., Hazout S. (2000). Bayesian probabilistic approach for predicting backbone structures in terms of protein blocks. *Proteins: Structure, Function, and Bioinformatics*.

[11] Zheng W.-M., Liu X. (2005). A protein structural alphabet and its substitution matrix CLESUM. *Transactions on Computational Systems Biology II*.

[12] Budowski-Tal I., Nov Y., Kolodny R. (2010). FragBag, an accurate representation of protein structure, retrieves structural neighbors from the entire PDB quickly and accurately. *Proceedings of the National Academy of Sciences of the United States of America*.

[13] Kleywegt G. J., Jones T. A. (1996). Phi/Psi-chology: ramachandran revisited. *Structure*.

[14] Faraggi E., Xue B., Zhou Y. (2009). Improving the prediction accuracy of residue solvent accessibility and real-value backbone torsion angles of proteins by guided-learning through a two-layer neural network. *Proteins: Structure, Function and Bioinformatics*.

[15] Shen Y., Delaglio F., Cornilescu G., Bax A. (2009). TALOS+: a hybrid method for predicting protein backbone torsion angles from NMR chemical shifts. *Journal of Biomolecular NMR*.

[16] Jones D. T., Buchan D. W. A., Cozzetto D., Pontil M. (2012). PSICOV: precise structural contact prediction using sparse inverse covariance estimation on large multiple sequence alignments. *Bioinformatics*.

[17] Vendruscolo M., Najmanovich R., Domany E. (1999). Protein folding in contact map space. *Physical Review Letters*.

[18] Holm L., Sander C. (1993). Protein structure comparison by alignment of distance matrices. *Journal of Molecular Biology*.

[19] Zhao F., Xu J. (2012). A position-specific distance-dependent statistical potential for protein structure and functional study. *Structure*.

[20] Joo K., Lee S. J., Lee J. (2012). Sann: solvent accessibility prediction of proteins by nearest neighbor method. *Proteins: Structure, Function and Bioinformatics*.

[21] Ma J., Wang S., Zhao F., Xu J. (2013). Protein threading using context-specific alignment potential. *Bioinformatics*.

[22] Ma J., Peng J., Wang S., Xu J. (2012). A conditional neural fields model for protein threading. *Bioinformatics*.

[23] Ma J., Wang S., Wang Z., Xu J. (2014). MRFalign: protein homology detection through alignment of markov random fields. *PLoS Computational Biology*.

[24] Benkert P., Künzli M., Schwede T. (2009). QMEAN server for protein model quality estimation. *Nucleic Acids Research*.

[25] Cheng J., Wang Z., Tegge A. N., Eickholt J. (2009). Prediction of global and local quality of CASP8 models by MULTICOM series. *Proteins: Structure, Function and Bioinformatics*.

[26] Kinjo A. R., Horimoto K., Nishikawa K. (2005). Predicting absolute contact numbers of native protein structure from amino acid sequence. *Proteins: Structure, Function and Genetics*.

[27] Kabakçioǧlu A., Kanter I., Vendruscolo M., Domany E. (2002). Statistical properties of contact vectors. *Physical Review E*.

[28] Tegge A. N., Wang Z., Eickholt J., Cheng J. (2009). NNcon: improved protein contact map prediction using 2D-recursive neural networks. *Nucleic Acids Research*.

[29] Holbrook S. R., Muskal S. M., Kim S.-H. (1990). Predicting surface exposure of amino acids from protein sequence. *Protein Engineering*.

[30] Rost B., Sander C. (1994). Conservation and prediction of solvent accessibility in protein families. *Proteins: Structure, Function and Genetics*.

[31] Ehrlich L., Reczko M., Bohr H., Wade R. C. (1998). Prediction of protein hydration sites from sequence by modular neural networks. *Protein Engineering*.

[32] Pollastri G., Baldi P., Fariselli P., Casadio R. (2002). Prediction of coordination number and relative solvent accessibility in proteins. *Proteins: Structure, Function, and Bioinformatics*.

[33] Ahmad S., Gromiha M. M. (2002). NETASA: neural network based prediction of solvent accessibility. *Bioinformatics*.

[34] Adamczak R., Porollo A., Meller J. (2004). Accurate prediction of solvent accessibility using neural networks-based regression. *Proteins: Structure, Function and Genetics*.

[35] Yuan Z., Burrage K., Mattick J. S. (2002). Prediction of protein solvent accessibility using support vector machines. *Proteins: Structure, Function and Genetics*.

[36] Kim H., Park H. (2004). Prediction of protein relative solvent accessibility with support vector machines and long-range interaction 3D local descriptor. *Proteins: Structure, Function and Genetics*.

[37] Nguyen M. N., Rajapakse J. C. (2005). Prediction of protein relative solvent accessibility with a two-stage SVM approach. *Proteins: Structure, Function and Genetics*.

[38] Thompson M. J., Goldstein R. A. (1996). Predicting solvent accessibility: higher accuracy using Bayesian statistics and optimized residue substitution classes. *Proteins: Structure, Function, and Genetics*.

[39] Sim J., Kim S.-Y., Lee J. (2005). Prediction of protein solvent accessibility using fuzzy k-nearest neighbor method. *Bioinformatics*.

[40] Ahmad S., Gromiha M. M., Sarai A. (2003). Real value prediction of solvent accessibility from amino acid sequence. *Proteins: Structure, Function and Genetics*.

[41] Garg A., Kaur H., Raghava G. P. S. (2005). Real value prediction of solvent accessibility in proteins using multiple sequence alignment and secondary structure. *Proteins: Structure, Function and Genetics*.

[42] Yuan Z., Huang B. (2004). Prediction of protein accessible surface areas by support vector regression. *Proteins: Structure, Function, and Bioinformatics*.

[43] Yuan Z. (2005). Better prediction of protein contact number using a support vector regression analysis of amino acid sequence. *BMC Bioinformatics*.

[44] Goldman N., Thorne J. L., Jones D. T. (1998). Assessing the impact of secondary structure and solvent accessibility on protein evolution. *Genetics*.

[45] Wang Z., Zhao F., Peng J., Xu J. (2011). Protein 8-class secondary structure prediction using conditional neural fields. *Proteomics*.

[46] Magnan C. N., Baldi P. (2014). SSpro/ACCpro 5: almost perfect prediction of protein secondary structure and relative solvent accessibility using profiles, machine learning and structural similarity. *Bioinformatics*.

[47] Peng J., Bo L., Xu J. (2009). Conditional neural fields. *Advances in Neural Information Processing Systems*.

[48] Wang S., Peng J., Xu J. (2011). Alignment of distantly related protein structures: algorithm, bound and implications to homology modeling. *Bioinformatics*.

[49] Zhao F., Peng J., Xu J. (2010). Fragment-free approach to protein folding using conditional neural fields. *Bioinformatics*.

[50] Källberg M., Wang H., Wang S. (2012). Template-based protein structure modeling using the RaptorX web server. *Nature Protocols*.

[51] Källberg M., Margaryan G., Wang S., Ma J., Xu J. (2014). RaptorX server: a resource for template-based protein structure modeling. *Protein Structure Prediction*.

[52] Dubchak I., Balasubramanian S., Wang S. (2014). An integrative computational approach for prioritization of genomic variants. *PLoS ONE*.

[53] Lafferty J., McCallum A., Pereira F. C. Conditional random fields: probabilistic models for segmenting and labeling sequence data.

[54] Collobert R., Weston J. A unified architecture for natural language processing: deep neural networks with multitask learning.

[55] Caruana R. (1998). *Multitask Learning*.

[56] Chothia C. (1976). The nature of the accessible and buried surfaces in proteins. *Journal of Molecular Biology*.

[57] Berman H. M., Westbrook J., Feng Z. (2000). The protein data bank. *Nucleic Acids Research*.

[58] Kozma D., Simon I., Tusnády G. E. (2013). PDBTM: protein data bank of transmembrane proteins after 8 years. *Nucleic Acids Research*.

[59] Jordan R. A., El-Manzalawy Y., Dobbs D., Honavar V. (2012). Predicting protein-protein interface residues using local surface structural similarity. *BMC Bioinformatics*.

[60] Sowdhamini R., Rufino S. D., Blundell T. L. (1996). A database of globular protein structural domains: clustering of representative family members into similar folds. *Folding and Design*.

[61] Moult J. (2005). A decade of CASP: progress, bottlenecks and prognosis in protein structure prediction. *Current Opinion in Structural Biology*.

[62] Adamczak R., Porollo A., Meller J. (2005). Combining prediction of secondary structure and solvent accessibility in proteins. *Proteins: Structure, Function and Genetics*.

[63] Cheng J., Randall A. Z., Sweredoski M. J., Baldi P. (2005). SCRATCH: a protein structure and structural feature prediction server. *Nucleic Acids Research*.

[64] Tseng Y. Y., Liang J. (2006). Estimation of amino acid residue substitution rates at local spatial regions and application in protein function inference: a Bayesian Monte Carlo approach. *Molecular Biology and Evolution*.

[65] Altschul S. F., Madden T. L., Schäffer A. A. (1997). Gapped BLAST and PSI-BLAST: a new generation of protein database search programs. *Nucleic Acids Research*.

[66] Söding J. (2005). Protein homology detection by HMM-HMM comparison. *Bioinformatics*.

[67] Biegert A., Söding J. (2009). Sequence context-specific profiles for homology searching. *Proceedings of the National Academy of Sciences of the United States of America*.

[68] Meiler J., Müller M., Zeidler A., Schmäschke F. (2001). Generation and evaluation of dimension-reduced amino acid parameter representations by artificial neural networks. *Journal of Molecular Modeling*.

[69] Duan M., Huang M., Ma C., Li L., Zhou Y. (2008). Position-specific residue preference features around the ends of helices and strands and a novel strategy for the prediction of secondary structures. *Protein Science*.

[70] Tan Y. H., Huang H., Kihara D. (2006). Statistical potential-based amino acid similarity matrices for aligning distantly related protein sequences. *Proteins: Structure, Function and Genetics*.

[71] Fei H., Huan J. (2013). Structured feature selection and task relationship inference for multi-task learning. *Knowledge and Information Systems*.

[72] Chapelle O., Shivaswamy P., Vadrevu S., Weinberger K., Ya Z., Tseng B. Multi-task learning for boosting with application to web search ranking.

[73] Chen J., Liu J., Ye J. (2012). Learning incoherent sparse and low-rank patterns from multiple tasks. *ACM Transactions on Knowledge Discovery from Data*.

[74] Liu J., Ji S., Ye J. Multi-task feature learning via efficient l_2, 1_-norm minimization.

[75] Qi Y., Oja M., Weston J., Noble W. S. (2012). A unified multitask architecture for predicting local protein properties. *PLoS ONE*.

[76] Wang S., Ma J., Peng J., Xu J. (2013). Protein structure alignment beyond spatial proximity. *Scientific Reports*.

[77] Wang S., Zheng W.-M. (2008). CLePAPS: fast pair alignment of protein structures based on conformational letters. *Journal of Bioinformatics and Computational Biology*.

[78] Wang S., Zheng W.-M. (2009). Fast multiple alignment of protein structures using conformational letter blocks. *The Open Bioinformatics Journal*.

[79] Ma J., Wang S. (2014). Algorithms, applications, and challenges of protein structure alignment. *Advances in Protein Chemistry and Structural Biology*.

[80] Zhang Y., Skolnick J. (2004). Scoring function for automated assessment of protein structure template quality. *Proteins: Structure, Function and Genetics*.

[81] Xu J., Zhang Y. (2010). How significant is a protein structure similarity with TM-score = 0.5?. *Bioinformatics*.

[82] Luo Q., Hamer R., Reinert G., Deane C. M. (2013). Local network patterns in protein-protein interfaces. *PLoS ONE*.

