# Supplementary material for: AcconPred: Predicting Solvent Accessibility and Contact Number Simultaneously by a Multitask Learning Framework under the Conditional Neural Fields Model
Source: Biomed Res Int. 2015 Aug 3;2015:678764. doi: 10.1155/2015/678764 (PMC4538422; doi:10.1155/2015/678764)
Supplement: Supplementary file 1 — Training/testing dataset as well as the CASP11 evaluation dataset could be found in the Supplementary Material. The CASP11 evaluation dataset contains all detailed results from SPINE-X, SANN, ACCpro5 and AcconPred. [file 678764.f1.zip › Supplmentary_Files/AcconPred_package_v1.00.tar.gz!/AcconPred_package_v1.00/util/BLAST/data/sequin.hlp]

 
Sequin help documentation


|  |  |
| --- | --- |
|  | Sequin Help Documentation |

|  |  |  |  |  |  |
| --- | --- | --- | --- | --- | --- |
| Sequin | Entrez | BLAST | OMIM | Taxonomy | Structure |

## Table of Contents

---

>Introduction
#Sequin is a program designed to aid in the submission of sequences to
the GenBank or EMBL sequence databases. It was written at the
National Center for Biotechnology Information, part of the National
Library of Medicine at the National Institutes of Health. This section
of the help document provides a basic overview of how to submit
sequences using the Sequin forms. Subsequent sections provide detailed
instructions for entering information on each form.
\*The Help Documentation
#The Sequin help documentation is available in both on-line and World
Wide Web (http://www.ncbi.nlm.nih.gov/Sequin/sequin.hlp.html) formats.
The text of the on-line version scrolls as you progress through the
Sequin forms. Specific words or phrases can be identified with the
"find" command at the top of the window. The on-line document can also
be saved as a text file, or printed directly to a printer. Click on the
window that contains the help documentation. Under the Sequin File
menu, choose Export Help... to save the documentation as a text file.
To print the documentation without saving it first, click on the help
window, and choose Print from the Sequin File menu.
\*Organization of Forms
#Information is entered into Sequin on a number of different forms. Each
form is made up of pages, which are indicated by folder tabs at the top
of the form. You can move to the desired page by clicking on the
appropriate folder tab. You can also move between pages of a form by
clicking on the "Next page" or "Prev page" buttons at the bottom of the
screen. You can move to the previous form or the next form by clicking
on the "Prev form" or "Next form" buttons on the first or last pages of
a form, respectively.
#There are numerous ways to enter information onto a page of a form,
including text fields, radio buttons, check boxes, scrolling boxes,
pop-up menus and spreadsheets.
#You may also use tables to import annotation of source information.
The formatting of these tables will be discussed below.
\*Overview of Sequin
#If you are using Sequin for the first time, you will be prompted to
fill out four forms: the Welcome to Sequin form, the Submitting
Authors Form, the Sequence Format form, and the Organism and Sequences
Form. After you have filled out these forms, a window will appear that
contains the Sequin record viewer. This viewer allows you to access
many other forms in which you can edit fields filled out in the three
initial forms, as well as add additional information. Detailed
instructions on how to fill out the forms and use the record viewer are
presented below.
>Welcome to Sequin Form
#First, indicate with one of the radio buttons whether you are
submitting the sequence to the GenBank or EMBL database. If you
are working on a sequence submission for the first time, click on
"Start New Submission". If you are modifying an existing submission
record, click on "Read Existing Record". If you would like to quit from
Sequin, click on "Quit Program".
#The "Network Configure" button will guide you through the set-up
process to run Sequin in its network-aware mode. Doing so allows
the program to exchange information with NCBI (GenBank) over the
Internet. Details are provided in the
instructions
later in the help documentation.
#You can also "Read Existing Record" to read in a FASTA-formatted sequence
file for analysis purposes. The sequence will be displayed in Sequin and can
be analyzed with tools such as CDD Search, but it should not be submitted
because it does not have the appropriate annotations.
#If you are running Sequin in its network-aware mode, you will see
another button labeled "Download from Entrez". This option allows you
to update an existing database record using Sequin. The record will be
downloaded from GenBank into Sequin using NCBI's Entrez retrieval
system. The contents of the record will appear in Sequin, and you can
edit them by updating the sequence or the annotations, as necessary. If
you do not see the button labeled "Download from Entrez" on the Welcome
to Sequin form, you are not running Sequin in its network-aware mode.
To make Sequin network-aware, see the
instructions
later in the help documentation.
#You can update only those records that you have submitted, not those
submitted by others. To update an existing record, first select which
of the databases you will be sending the update to. This should be the
database to which the original record was submitted. If you do not
know which database to use, send the record to GenBank and the NCBI
staff will forward it to the appropriate database. Next, click on the
button "Download from Entrez". Enter the nucleotide Accession number or
GI of the sequence on the first form. Then enter "yes" if you are
planning to submit the record as an update to one of the databases.
Fill out the Submitting Authors form.
Instructions
for this form are found in the Sequin help documentation under "Edit
Submitter Info" under the Sequin File menu. The record will then open
in the record viewer. Explanations of how to add annotations or update
sequences are presented in the documentation entitled
"Editing the record"
and
Sequence Editor
respectively. You will not see the Submitting Authors Form, the
Sequence Format Form, or the Organism and Sequences Form. Note that
updates, as well as new records, must be emailed to the appropriate
database. Sequin does not support direct submission of records over the
Internet.
#Additional configuration options are available under the Misc menu.
You can toggle between the stand-alone and network-aware modes of
Sequin. The default mode of Sequin, which is sufficient for most
sequence submissions, is stand-alone. In its network-aware mode, Sequin
can exchange data with NCBI and, for example, retrieve sequences
from Entrez and perform Taxonomy searches. The network-aware mode of
Sequin is described in detail in the
Net Configure
section below. You can also start the NCBI DeskTop, which is for
advanced Sequin users only.
>Submitting Authors Form
#Information from this form will be used as a citation for the sequence
entry itself. It can contain the same information found in citations
associated with the formal publication of the sequence.
#On the bottom of each form are two buttons. Click "Prev form" (first
page in a form) or "Prev page" (subsequent pages in a form) to go to the
previous form or page. Click "Next Form" (last page on a form) or "Next
Page" (earlier pages on a form) to move to the next form or page.
#Form pages can also be saved individually by using the "Export" function
under the File menu. If you are processing multiple submissions, you
can use the "Import" function under the File menu to paste previously
entered information directly on the page.
#The Contact, Authors, and Affiliation pages can be saved as a block so
that you can use this information for your next submission. For your
first Sequin submission, fill in the requested information on the
Submitting Authors form and proceed with the preparation of the
submission. Choose Export Submitter Info under the File menu to export
this to a file. You can then import this information in subsequent
submissions using the Import Submitter Info in the File menu. You will
need to fill in the manuscript title for each submission however.
\*Submission Page
\*\*When May We Release Your Sequence Record?
#Please select one of the two radio buttons. If you select "Immediately
After Processing", the entry will be released to the public after the
database staff has added it to the database. If you select "Release
Date", fields will appear in which you can indicate the date on which
the sequences should be released to the public. The submission will
then be held back until formal publication of the sequence or GenBank
Accession number, or until the release date, whichever comes first. The
maximum hold time is five years.
\*\*Tentative Title for Manuscript
#Please enter a title that appropriately describes the sequence entry.
Later in the submission process, you will have the opportunity to change
this information and add details for published or in press references.
\*Contact Page
#Please enter the name, telephone and fax numbers, and email address of
the person who is submitting the sequence. This is the person who will
be contacted regarding the sequence submission. The phone, fax, and
email address will not be visible in the database record, but are
essential for contact by the database staff.
\*Authors Page
#Please enter the names of the people who should receive scientific
credit for the generation of sequences in this entry. The person on
the Contact page is automatically listed as the first author. This
information can be changed if necessary. The author names should be
entered in the order first name, middle initial, surname. You can add
as many authors to this page as you wish. After you type in the name
of the third author, the box becomes a spreadsheet, and you can scroll
down to the next line by using the space bar. The consortium box
should only be used for consortium names, not institute or department
names.
\*Affiliation Page
#Please enter information about the principal institution where the
sequencing was performed. This is not necessarily the same as the
workplace of the person described on the Contact page. This information
will show up in the reference section of the record, with the title
Direct Submission.
>Preparing the Sequences Form
#Use the radio buttons to select the type of dialogs used to prepare your
submission. The NormalSubmission Dialog can be used for all types of
submissions. Instructions continue with the
Sequence Format Form
.
#The Submission Wizards should only be used for virus sequences, uncultured
samples (environmental sequences), or rRNA-ITS-IGS sequences from cultured
bacteria and fungi or eukaryotic samples. These wizards will guide you to provide all
required source information for these types of submissions and will assist
with annotation.
>Wizard Import Nucleotide FASTA
#The requirements for samples to be submitted using the Submission Wizard
are listed in the dialog box.
#Use the Import Nucleotide FASTA button to import your properly formatted
FASTA file
. You will see a window containing information about the imported
sequence(s). Please check the number of sequences, Sequence IDs (SeqIDs)
and length of each sequence to make sure they are correct.
>Sequencing Method
#If you are submitting over 500 sequences or your sequences were generated
using next-generation sequencing technology, the information in this form is
required. Use the check boxes at the top of the form to select the sequencing
technology type(s) used to obtain the sequences. Multiple types can be
selected, if appropriate. If you used technology that is not listed in the
form, please select other and use the free text box to provide the
information. After selecting the sequencing technology, select the radio
button to indicate if your sequences are raw sequence reads or sequence
assemblies. If you are submitting assemblies using next-generation sequencing
technology, the name of the assembly program and program version or date the
assemblies were made are required in the free text boxes.
>Virus Wizard Type of Virus
#Use this page to select the type of virus sequence(s) you are submitting. If
you are not submitting any of the specific types listed or you are submitting
a mixed set of sequences from different viruses, select the "Not listed above
or mixed set of different viruses" radio button.
>rRNA-ITS-IGS Wizard Type of Source
#Use this page to select the type of organism from which your sequences were obtained.
>Wizard Source Organism Information
#All wizard submissions have specific source requirements. General information
for inputting source information will be followed by specific requirements for
each type of wizard submission.
#If you did not add source information using the
FASTA definition line
you must do so in this form. If you entered source information in the FASTA
definition lines, minimal input will be necessary on this form. However, if
there are any issues with the imported information, they will be listed on the
right side of the form. Please review the problems and edit the information.
You can display only the rows with problems by selecting the "Show only
sequences with errors" radio button.
#You can set the same source qualifier value for all sequences by filling in
the top row of boxes and using the appropriate Apply button. You can also use
the Copy from SeqID button to apply the sequence IDs to the qualifier
indicated in the table if this information was used as the sequences ID in the
original FASTA file.
#The required information can also be directly typed into the spreadsheet in
the middle of the form or imported from a tab-delimited table using the Import
Source Table button. Click on the Source Table Help button to open a text
dialog with information on making a tab-delimited source table.
#If you have made any changes on this form, please use the Recheck Errors
button to validate the new information. Any problems will be listed on
the right side of the spreadsheet. You can use the Show only sequences
with errors radio button to list only those sequences that did not pass
the validation.
A complete list of available source
modifiers
and their format is available.
\*Virus Wizard Source Information
Each type of virus submission has specific requirments. In addition to the
specific requirements listed below, you will need to provide unique source
information (such as unique strain or isolate names/IDs) for each sequence if
all of your sequences are from the same gene/region.
\*\*Norovirus, Sapovirus (Caliciviridae)
#Norovirus or Sapovirus (Caliciviridae) sequences must list the organism name,
isolate, collection date, country, and host or isolation source. Please use
the Add isolation-source button if the source of the virus is better described
as an isolation source rather than host. Optional modifiers can be added to
provide additional information.
\*\*Foot-and-mouth disease virus
#Foot-and-mouth disease virus sequences must list the organism name and
isolate. Please use the other qualifiers in the table to provide additional
information about the source.
\*\*Influenza virus
#Influenza virus sequences must list the organism name, properly formatted
strain, collection date and host or isolation source. Influenza A viruses also
must list the serotype. Please use the other qualifiers in the table to
provide additional information about the source.
\*\*Rotavirus
#Rotavirus sequences must list the organism name, isolate, collection date,
country, and host or isolation source. Please use the Add isolation-source
button if the source of the virus is better described as an isolation source
rather than host. Optional modifiers can be added to provide additional
information.
\*\*Not listed above or mixed set of different viruses
#All other virus sequences must list the organism name and isolate. Please use
the other qualifiers in the table to provide additional information about the
source.
\*Uncultured Sample Wizard Source Information
#All sequences must have an organism name, isolation source or host, and unique
clone name. Optional modifiers can be added to provide additional information,
if known. The organism name should not contain the entire lineage information.
See the NCBI
Taxonomy Browser
 for more information about the organism names.
#The clone name is used for both traditional clones and PCR product sample IDs.
The clone names must be unique within the submission. In addition, information
about the environment from which the sequences were isolated should be
supplied within the isolation source field. If the source organism was
isolated from within a host organism, this should be supplied in the host
field.
\*rRNA-ITS-IGS Wizard Source Information
#All sequences must have an organism name. Bacterial, archaeal, and fungal
sequences must have unique strain names. Sequences from all other organisms
must have either unique organism names or a combination of unique organism
names and isolate names.
>Virus Wizard Molecule Information
#Use this page to select the molecule type that was isolated and sequenced in
your experiment. The topology of the molecule can also be changed in this
window. Only set the topology to circular if you are submitting a complete,
circular viral genome or segment. Single genes or fragments of viral genomes
should not be set to a circular topology.
#If you selected mRNA as the molecule type you will be prompted for more
information about your samples.
>Virus Wizard Annotation
#Use the radio buttons to select the option that best describes the sequences.
After completing all dialogs for each section, you will be directed to leave
the Wizard and transferred to the
record viewer
. You must do so to complete your submission. However, you cannot return to the Wizard once you have exited.
\*Single coding region across the entire sequence
#Select this option if the sequences contain a single coding region across the
entire length of all of the sequences. Once you have selected this button, a
new dialog will appear with text boxes to input the protein name, protein
description, gene symbol and comments. Only the protein name is required,
other fields are optional. If the coding region is partial, check the
appropriate 5' or 3' boxes near the top of the dialog as appropriate.
\*Single non-coding feature across the entire sequence
#Select this option if the sequences contain a single non-coding feature across
the entire length of all of the sequences. Once of you have selected this
button, a new dialog will appear listing common types of non-coding features.
Select the appropriate radio button. If none of the choices listed are
appropriate, select Something else and a free text box will appear for you to
type a description of what the sequences contain.
\*Multiple features per sequence (coding regions, LTRs, etc.)
#Select this option if the sequences contain more than one feature and you know
the nucleotide spans of each feature. Once this option is selected, you will
be prompted to exit the wizard and the record viewer will open. A text dialog
will open with instructions for importing a five-column, tab-delimited feature
table containing the feature locations. You may also add the feature
annotation using the menu options in the record viewer.
>Uncultured Sample Wizard Primer Type
#Use this page to select the type of primers used in PCR amplification of
the samples.
>Uncultured Sample Wizard Annotation
#Use the radio buttons to select the option that best describes the
sequences. After completing all dialogs for each section, you will be
directed to leave the Wizard and transferred to the
record viewer
. You must do so to complete your submission. However, you cannot return
to the Wizard once you have exited.
\*Single rRNA, ITS, or IGS
#Select this option if the sequences contain a single ribosomal RNA, one
internal transcribed spacer or one intergenic spacer across the entire
sequence. Once you have selected this button, a new dialog will appear
with radio buttons to select the type of organism from which the
sequences were derived. Once the organism type is selected, a dialog
listing common types of rRNA, ITS or IGS will appear. Select the
appropriate radio button. If none of the choices are appropriate,
select Something else and type in a description of what the sequences
contain.
\*Multiple rRNA, ITS, or IGS regions where spans are unknown
#Select this option if the sequences contain more than one ribosomal RNA,
internal transcribed spacer(s) and/or intergenic spacer and you are
unsure of the nucleotide locations of each feature. Once you have
selected this button, a new dialog will appear with radio buttons to
select the type of organism from which the sequences were derived. Once
the organism type is selected, a dialog listing common types of rRNA,
ITS and IGS will appear. Select the appropriate radio button. If none
of the choices are appropriate, select Something else and type in a
description of what the sequences contain.
\*Multiple rRNA, ITS, or IGS where spans are known
#Select this option if the sequences contain more than one ribosomal RNA,
internal transcribed spacer(s) and/or intergenic spacer and you know the
nucleotide spans of each feature. Once this option is selected, you will be
prompted to exit the wizard and the record viewer will open. A text dialog
will open with instructions for importing a five-column, tab-delimited table
containing the feature locations. You may also apply annotation using the
Annotate menu options in the record viewer.
\*Coding Region (CDS)
#Select this option if the sequences encode a single protein across the
entire length of the sequence. Once you have selected this button, a
new dialog will appear with text boxes to input the protein name,
protein description, gene symbol and comments. Only the protein name is
required, other fields are optional. If the coding region is partial,
check the appropriate 5' or 3' boxes near the top of the dialog as
appropriate.
\*Something else, multiple features
#Select this option if the sequences do not contain one of the feature types
listed in the annotation dialog or you wish to apply annotation in the record
viewer. Upon selecting this option, you will be prompted to exit the wizard
and the record viewer will open. A text dialog will open with instructions for
importing a five-column, tab-delimited table. You may apply annotation with a
five-column, tab-delimited table or using the Annotate menu options in the
record viewer. Upon selecting this option you will be prompted to exit the
wizard and the record viewer will open.
>rRNA-ITS-IGS Wizard Genome
#This dialog will only appear if you selected Cultured Fungus or Something else
in the rRNA-ITS-IGS Wizard Type of Source form. Use this page to select the
genome from which your sequences are derived. If your sequences are derived
from nuclear genomic DNA, select nuclear. If your sequences are derived from
an alternative location (i.e. organelle genome), select the
appropriate
location
in this dialog.
>rRNA-ITS-IGS Wizard Annotation
#Use the radio buttons to select the option that best describes the
sequences. After completing all dialogs for each section, you will be
directed to leave the wizard and transferred to the
record viewer
. You must exit the wizard to complete your submission. However, you cannot return
to the Wizard once you have exited.
\*Single rRNA or IGS/Single rRNA or ITS/Single rRNA
Select this option if the sequences contain a single ribosomal RNA, one
internal transcribed spacer or one intergenic spacer across the entire
sequence. Once you have selected this button, a new dialog listing common
types of rRNA, ITS or IGS will appear. Select the appropriate radio button.
If none of the choices are appropriate, select Something else and type in
a description of what the sequences contain.
\*Multiple rRNA or IGS where spans are unknown/Multiple rRNA or ITS where spans are unknown
#Select this option if the sequences contain more than one ribosomal RNA,
internal transcribed spacer(s) and/or intergenic spacer and you are
unsure of the nucleotide locations of each feature. Once you have
selected this button, a new dialog listing common types of rRNA,
ITS and IGS will appear. Select the appropriate radio button. If none
of the choices are appropriate, select Something else and type in a
description of what the sequences contain.
\*Multiple rRNA or IGS where spans are known/Multiple rRNA or ITS where spans are known
#Select this option if the sequences contain more than one ribosomal RNA,
internal transcribed spacer(s) and/or intergenic spacer and you know the
nucleotide spans of each feature. Once this option is selected, you will be
prompted to exit the wizard and the record viewer will open. A text dialog
will open with instructions for importing a five-column, tab-delimited table
containing the feature locations. You may also apply annotation using the
Annotate menu options in the record viewer.
\*Something else
#Select this option if the sequences do not contain one of the feature types
listed in the annotation dialog or you wish to apply annotation in the record
viewer. Upon selecting this option, you will be prompted to exit the wizard
and the record viewer will open. A text dialog will open with instructions for
importing a five-column, tab-delimited table. You may apply annotation with a
five-column, tab-delimited table or using the Annotate menu options in the
record viewer. Upon selecting this option you will be prompted to exit the
wizard and the record viewer will open.
>Wizard rRNA Chimera Checking
#If you selected the annotation of 16S ribosomal RNA sequences from
bacteria or archaea, an additional dialog will appear asking if the
sequences have been screened with a chimera check program. If you have
used a chimera check program, please provide the name and version if
applicable. Note that BLAST is not a chimera check program. If you
have screened the sequences for chimeras, please be sure to remove all
suspected chimera sequences before submission.
>Sequence Format Form
#Use this form to indicate the type, format and category of sequence
you are submitting.
#Sequin can process single nucleotide sequences, gapped sequences and
sets of related sequences. If the sequences are related in terms of
coming from the same publication, or the same organism, they may be
candidates for a Batch submission. Biologically related sequences may
be classified as environmental samples, population, phylogenetic, or
mutation sets as appropriate. In all cases, although the sequences are
handled as a single submission, each sequence in a set will receive its
own database Accession number and can be annotated independently.
#Sequin can display the alignments of sequences that are submitted as
part of an aligned phylogenetic, population, mutation set, or
environmental samples. Such sequences can be submitted in FASTA,
Contiguous (FASTA+GAP, NEXUS, MACAW), or Interleaved (PHYLIP, NEXUS)
formats. If the sequences are in FASTA format, Sequin can generate an
alignment. If the sequences have already been aligned in FASTA+GAP,
PHYLIP, MACAW, or NEXUS, Sequin will not change the alignment. If one
of the sequences in your alignment is already present in the
GenBank/EMBL/DDBJ database, you must mark that sequence so that it does
not receive a new Accession number. Instead of supplying that sequence
with a new Sequence Identifier, give it the identifier accU12345, where
U12345 is the Accession number of the sequence.
#Single sequences, gapped sequences, and batch submissions must be
submitted in FASTA format.
\*Submission Type
#Use the radio buttons to indicate which of the following types of
submissions you are creating:
#-Single sequence: a single mRNA or genomic DNA sequence. If you are
submitting multiple sequences from the same publication, consider a
Batch Submission. If you decide to submit multiple Sequin files, each
with one or more sequences, please send each file in a separate email
message.
#-Gapped sequence: a single, non-contiguous mRNA or genomic DNA sequence.
A gapped sequence contains specified gaps of know or unknown length
where the exact nucleotide sequence has not been determined. The FASTA
format for gapped sequences is slightly different and is explained
below.
#-Population study: a set of sequences that were derived by sequencing
the same gene from different isolates of the same organism.
#-Phylogenetic study: a set of sequences that were derived by sequencing
the same gene from different organisms.
#-Mutation study: a set of sequences that were derived by sequencing
multiple mutations of a single gene.
#-Environmental samples: a set of sequences that were derived by
sequencing the same gene from a population of unclassified or unknown
organisms.
#-Batch submission: a set of related sequences that are not part of a
population, mutation, or phylogenetic study. The sequences should be
related in some way, such as coming from the same publication or
organism. You should plan that all sequences will be released to the
public on the same date.
\*Sequence Data Format
#If you are submitting a single or gapped sequence, or a batch
submission, your sequence must be in FASTA format, described
below. If you are submitting a set of sequences as part of a
population, phylogenetic, or mutation study, you have a choice of
sequence formats. You may submit the set as individual sequences in
FASTA format. Alternatively, you can submit the sequences as part of an
alignment. Sequin currently accepts the alignment formats FASTA+GAP,
PHYLIP, MACAW, NEXUS Interleaved, and NEXUS Contiguous.
\*Submission Category
#Use the radio buttons to indicate whether your sequence corresponds to
an original submission or a third-party annotation submission. If you
have directly sequenced the nucleotide sequence in your laboratory,
your submission would be considered an original submission.
#If you have downloaded the sequence from GenBank and added to it your
own annotations, your entry may be eligible for submission to the
Third-Party Annotation Database
(TPA)
.
#In order to be released into the TPA database, the sequence must appear
in a peer-reviewed publication in a biological journal. If you select
this option, a pop-up box will appear upon the completion of the
Sequence Format form. You must provide some description of the
biological experiments used as evidence for the annotation of your TPA
submission in this box.
#You will be asked later in the submission process to provide the GenBank
Accession number(s) of the primary sequence(s) from which your TPA
submission was derived.
>Organism and Sequences Form
#This form is made up of four pages. If your sequences are imported as
properly formatted FASTA files, there will be minimum input necessary
in these pages.
>FASTA Format for Nucleotide Sequences
#In FASTA format the line before the nucleotide sequence, called the
FASTA definition line, must begin with a carat (">"), followed by a
unique SeqID (sequence identifier). The SeqID must be unique for each
nucleotide sequence and should not contain any spaces. Please limit
the SeqID to 25 characters or less. Use of brackets
("[]") in the SeqID is also prohibited. The identifier will be
replaced with an Accession number by the database staff when your
submission is processed.
#Information about the source organism from which the sequence was
obtained follows the SeqID and must be in the format [modifier=text].
Do not put spaces around the "=". At minimum, the scientific name of
the organism should be included. Optional modifiers can be added to
provide additional information. A complete list of available source
modifiers
and their format is available.
#The final optional component of the FASTA definition line is the
sequence title, which will be used as the DEFINITION field in the final
flatfile. The title should contain a brief description of the
sequence. There is a preferred format for nucleotide and protein
titles. The provided title may be changed to the proper format by the
database staff during processing.
#Note in all cases, the FASTA definition line must not contain any hard
returns. All information must be on a single line of text. If you
have trouble importing your FASTA sequences, please double check that
no returns were added to the FASTA definition line by your editing
software.
#Examples of properly formatted FASTA definition lines for nucleotide
sequences are:
 ```` ```
>Seq1 [organism=Mus musculus] [strain=C57BL/6] Mus musculus neuropilin 1 (Nrp1) mRNA, complete cds.
``` ```` 
 ```` ```
>ABCD [organism=Plasmodium falciparum] [isolate=ABCD] Plasmodium falciparum isolate ABCD merozoite surface protein 2 (msp2) gene, partial cds.
``` ```` 
 ```` ```
>DNA.new [organism=Homo sapiens] [chromosome=17] [map=17q21] [moltype=mRNA] Homo sapiens breast and ovarian cancer susceptibility protein (BRCA1) mRNA, complete cds.
``` ```` 
#The line after the FASTA definition line begins the nucleotide
sequence. Unlike the FASTA definition line, the nucleotide sequence
itself can contain returns. It is recommended that each line of
sequence be no longer than 80 characters. Please only use IUPAC
symbols within the nucleotide sequence. For sequences that are not
contained within an alignment, do not use "?" or "-" characters. These
will be stripped from the sequence. Use the IUPAC approved symbol "N"
for ambiguous characters instead.
#A single file containing multiple FASTA sequences can be imported into
Sequin in order to create a
Batch Submission
. Make sure that the FASTA definition line for each sequence is
formatted as above.
#If the FASTA definition line is not properly formatted a pop-up box
will appear upon importing the nucleotide FASTA. The top box in this
pop-up will list any errors in the FASTA definition lines, including
missing SeqIDs, duplicate SeqIDs for different sequences, or improperly
formatted modifiers. You can add or edit this information in the
spreadsheet provided. The toggle at the bottom of the pop-up allows
you to select whether all sequences or only those with errors are
listed in the spreadsheet above. After making changes, click on Refresh
Error List to ensure that all errors have been corrected. You must
correct any errors involving the SeqID in order to proceed with your
submission.
\*FASTA Format for Gapped Sequence
#The FASTA definition line for a gapped sequence follows the same format
as above. To indicate a gap within the sequence, enter a hard return
within the sequence at the point of the gap, then insert an extra line
starting with a carat (">") and a question mark ("?"). If the gap size
is unknown, enter "unk100" after the question mark. If the gap size is
known, enter the length of the gap after the question mark. For
example,
!>Dobi [organism=Canis familiaris] [breed=Doberman pinscher]
!AAATGCATGGGTAAAAGTAGTAGAAGAGAAGGCTTTTAGCCCAGAAGTAATACCCATGTTTTCAGCATTA
!GGAAAAAGGGCTGTTG
!>?unk100
!TGGATGACAGAAACCTTGTTGGTCCAAAATGCAAACCCAGATKGTAAGACCATTTTAAAAGCATTGGGTC
!TTAGAAATAGGGCAACACAGAACAAAAAT
!>?234
!AAAAATAAAAGCATTAGTAGAAATTTGTACAGAACTGGAAAAGGAAGGAAAAATTTCAAAAATTGGGCCT
!GAAAACCCATACAATACTCCGGG
will generate a sequence containing two gaps. The first gap is of
unknown length, the second is 234 nucleotides long.
\*FASTA+GAP Format for Aligned Nucleotide Sequences
#A number of programs output sets of aligned sequences in FASTA format.
Frequently, to align these sequences, gaps must be inserted. The
default alignment settings should correctly interpret gap and ambiguous
characters in most cases. If Sequin can not read your alignment, you
may need to change these settings using the Optional Alignment Settings
button on the
Nucleotide Page
form. Each sequence, including gaps, must be the same length. The
gaps will only show up in the alignment, not in the individual sequence
in the database.
#Sequences in FASTA+GAP format resemble FASTA sequences. The previous
section on
FASTA Format for Nucleotide Sequences
has instructions for formatting FASTA sequences. If one of the
sequences in your alignment is already present in the GenBank/EMBL/DDBJ
database, you must mark that sequence so that it does not receive a new
Accession number. To do this, use a SeqID in the format accU12345,
where U12345 is the Accession number of the pre-existing sequence. All
sequences in FASTA+GAP format should be in the same file.
#The following is an example of FASTA+GAP format:
!>A-0V-1-A [organism=Gallus gallus] [clone=C]
!TCACTCTTTGGCAACGACCCGTCGTCATAATAAAGATAGAGGGGCAACTAAAGGAAGCTCTA
!TTAGATACAGGAGCAGATGATACAGTATTAGAAGAAAT
!
!>A-0V-2-A [organism=Drosophila melanogaster] [strain=D]
!TCACTCTTTGGCAAC---GCGTCGTCACAATAAAGATAGAGGGGCAACTAAAGGAAGCTCTA
!TTAGATACAGGAGCAGATGATACAGTATTAGAAGAAAT
!
!>A-0V-3-A [organism=Caenorhabditis elegans] [strain=E]
!TCACTCTTTGGCAAC---GCGTCGTCACAATAAAGATAGAGGGGCAACTAAAGGAAGCTCTA
!TTAGATACAGGAGCAGATGATACAGTATTAGAAGAAAT
!
!>A-0V-4-A [organism=Rattus norvegicus] [strain=F]
!TCACTCTTTGGCAACGACCCGTCGTCACAATAAAGATAGAGGGGCAACTAAAGGAAGCTCTA
!TTAGATACAGGAGCAGATGATACAGTATTAGAAGAAAT
!
!>A-0V-7-A [organism=Aspergillus nidulans] [strain=G]
!TCACTCTTTGGCAACGACCAGTCGTCACAATAAAGATAGAGGGGCAACTAAAGGAAGCTCTA
!TTAGATACAGGAGCAGATGATACAGTATTAGAAGAAAT
\*PHYLIP Format for Aligned Nucleotide Sequences
#A number of programs output sets of aligned sequences in PHYLIP format.
#The following is an example of PHYLIP format.
! 5 100
!A-0V-1-A TCACTCTTTG GCAACGACCC GTCGTCATAA TAAAGATAGA GGGGCAACTA
!A-0V-2-A TCACTCTTTG GCAAC---GC GTCGTCACAA TAAAGATAGA GGGGCAACTA
!A-0V-3-A TCACTCTTTG GCAAC---GC GTCGTCACAA TAAAGATAGA GGGGCAACTA
!A-0V-4-A TCACTCTTTG GCAACGACCC GTCGTCACAA TAAAGATAGA GGGGCAACTA
!A-0V-7-A TCACTCTTTG GCAACGACCA GTCGTCACAA TAAAGATAGA GGGGCAACTA
!
!
! AAGGAAGCTC TATTAGATAC AGGAGCAGAT GATACAGTAT TAGAAGAAAT
! AAGGAAGCTC TATTAGATAC AGGAGCAGAT GATACAGTAT TAGAAGAAAT
! AAGGAAGCTC TATTAGATAC AGGAGCAGAT GATACAGTAT TAGAAGAAAT
! AAGGAAGCTC TATTAGATAC AGGAGCAGAT GATACAGTAT TAGAAGAAAT
! AAGGAAGCTC TATTAGATAC AGGAGCAGAT GATACAGTAT TAGAAGAAAT
#In this example, the first line indicates that there are 5 sequences,
each with 100 nt of sequence. The following five lines contain the
Sequence IDs, followed by the sequences. Specifically, the sequence
identifier for the first sequence is A-0V-1-A. Note that subsequent
blocks of sequence do not contain the Sequence ID. If one of the
sequences in your alignment is already present in the GenBank/EMBL/DDBJ
database, you must mark that sequence so that it does not receive a new
Accession number. To do this, use a SeqID in the format accU12345,
where U12345 is the Accession number of the pre-existing sequence.
#The default alignment settings should correctly interpret gap and
ambiguous characters in most cases. If Sequin can not read your
alignment, you may need to change these settings using the Optional
Alignment Settings button on the
Nucleotide Page
form.
#You can modify the PHYLIP format so that Sequin can
determine the correct organism and any other modifiers for each
sequence. An example of such modifications are below in the section on
Source Modifiers for PHYLIP and NEXUS
.
#Alternatively, you can leave your sequence alignment in
standard PHYLIP format and enter the organism, strain, chromosome, etc.
information on the following
Source Modifers form
.
\*NEXUS Format for Aligned Nucleotide Sequences
#A number of programs output sets of aligned sequences in one of two
NEXUS formats, NEXUS Interleaved and NEXUS Contiguous.
#NEXUS files can contain ? for "missing" at the 5' and 3' ends of
sequences, as long as this parameter is properly defined within the
header of the NEXUS file.
#The following is an example of NEXUS Interleaved format.
!#NEXUS
!
!begin data;
! dimensions ntax=5 nchar=100;
! format datatype=dna missing=? gap=- interleave;
! matrix
!
!A-0V-1-A TCACTCTTTG GCAACGACCC GTCGTCATAA TAAAGATAGA GGGGCAACTA
!A-0V-2-A TCACTCTTTG GCAAC---GC GTCGTCACAA TAAAGATAGA GGGGCAACTA
!A-0V-3-A TCACTCTTTG GCAAC---GC GTCGTCACAA TAAAGATAGA GGGGCAACTA
!A-0V-4-A TCACTCTTTG GCAACGACCC GTCGTCACAA T????ATAGA GGGGCAACTA
!A-0V-7-A TCACTCTTTG GCAACGACCA GTCGTCACAA TAAAGATAGA GGGGCAACTA
!
!
!A-0V-1-A AAGGAAGCTC TATTAGATAC AGGAGCAGAT GATACAGTAT TAGAAGAAAT
!A-0V-2-A AAGGAAGCTC TATTAGATAC AGGAGCAGAT GATACAGTAT TAGAAGAAAT
!A-0V-3-A AAGGAAGCTC TATTAGATAC AGGAGCAGAT GATACAGTAT TAGAAGAAAT
!A-0V-4-A AAGGAAGCTC TATTAGATAC AGGAGCAGAT GATACAGTAT TAGAAGAAAT
!A-0V-7-A AAGGAAGCTC TATTAGATAC AGGAGCAGAT GATACAGTAT TAGAAGAAAT
#In this example, the first few lines provide information about the data
in the sequence alignment. The following five lines contain the
Sequence IDs, followed by the sequences. Specifically, the sequence
identifier for the first sequence is A-0V-1-A. Note that subsequent
blocks of sequence also contain the Sequence ID. If one of the
sequences in your alignment is already present in the GenBank/EMBL/DDBJ
database, you must mark that sequence so that it does not receive a new
Accession number. To do this, use a SeqID in the format accU12345,
where U12345 is the Accession number of the pre-existing sequence.
Also, Sequin will replace the "?" characters in the sequences with "N"s
since they are defined as "missing" data in the header. The default
alignment settings should correctly interpret gap and ambiguous
characters in most cases. If Sequin can not read your alignment, you
may need to change these settings using the Optional Alignment Settings
button on the
Nucleotide Page
form.
#You can modify either NEXUS format so that Sequin can
determine the correct organism and any other modifiers for each
sequence. An example of such modifications are below in the section on
Source Modifiers for PHYLIP and NEXUS
.
#Alternatively, you can leave your sequence alignment in
standard NEXUS format and enter the organism, strain, chromosome, etc.
information on the following
Source Modifers form
.
#The following is an example of NEXUS Contiguous format.
!#NEXUS
!BEGIN DATA;
!DIMENSIONS NTAX=5 NCHAR=100;
!FORMAT MISSING=? GAP=- DATATYPE=DNA ;
!MATRIX
!
!A-0V-1-A
!TCACTCTTTGGCAACGACCCGTCGTCATAATAAAGATAGAGGGGCAACTAAAGGAAGCTCTA
!TTAGATACAGGAGCAGATGATACAGTATTAGAAGAAAT
!
!A-0V-2-A
!TCACTCTTTGGCAAC---GCGTCGTCACAATAAAGATAGAGGGGCAACTAAAGGAAGCTCTA
!TTAGATACAGGAGCAGATGATACAGTATTAGAAGAAAT
!
!A-0V-3-A
!TCACTCTTTGGCAAC---GCGTCGTCACAATAAAGATAGAGGGGCAACTAAAGGAAGCTCTA
!TTAGATACAGGAGCAGATGATACAGTATTAGAAGAAAT
!
!A-0V-4-A
!TCACTCTTTGGCAACGACCCGTCGTCACAATAAAGATAGAGGGGCAACTAAAGGAAGCTCTA
!TTAGATACAGGAGCAGATGATACAGTATTAGAAGAAAT
!
!A-0V-7-A
!TCACTCTTTGGCAACGACCAGTCGTCACAATAAAGATAGAGGGGCAACTAAAGGAAGCTCTA
!TTAGATACAGGAGCAGATGATACAGTATTAGAAGAAAT
#In this example, the first few lines provide information about the data
in the sequence alignment. The following five lines contain the
Sequence IDs, followed by the sequences. Specifically, the sequence
identifier for the first sequence is A-0V-1-A. Note that subsequent
blocks of sequence also contain the Sequence ID. If one of the
sequences in your alignment is already present in the GenBank/EMBL/D
DBJ database, you must mark that sequence so that it does not receive a
new Accession number. To do this, use a SeqID in the format accU12345,
where U12345 is the Accession number of the pre-existing sequence.
#You can modify either NEXUS format so that Sequin can
determine the correct organism and any other modifiers for each
sequence. An example of such modifications are below in the section on
Source Modifiers for PHYLIP and NEXUS
.
#Alternatively, you can leave your sequence alignment in
standard NEXUS format and enter the organism, strain, chromosome, etc.
information on the following
Source Modifers form
.
\*\*Source Modifiers for PHYLIP and NEXUS
#You can modify the PHYLIP or NEXUS formats so that Sequin can determine
the correct organism and any other modifiers for each sequence by
adding lines at the end of the file. The first line applies to the
first sequence, the second line to the second sequence, and so on. You
must have one line for each sequence. These inserted lines contain
modifiers formatted like in the FASTA definition line, but do not begin
with a SeqID. Instead, the SeqID is present at the beginning of the
sequence lines as shown above.
#Each of the initial lines starts with the character ">". The
scientific organism name follows in brackets. Optional modifiers also
follow in brackets. For further information on the data that can go in
the lines preceding the sequences, see the instructions entitled "FASTA
Format for Nucleotide Sequences",
above.
#The following lines indicating the organisms and strain of each sequence
would follow immediately after the sequence in the PHYLIP and NEXUS
examples, above.
!;
!END;
!
!begin ncbi;
!sequin
!>[organism=Gallus gallus] [clone=C]
!>[organism=Drosophila melanogaster] [strain=D]
!>[organism=Caenorhabditis elegans] [strain=E]
!>[organism=Rattus norvegicus] [strain=F]
!>[organism=Aspergillus nidulans] [strain=G]
!;
!end;
#The number of lines of source information must exactly match the number
of sequences provided. Complete examples can be found in the
Alignment Formats
section of the Sequin Quick Guide.
#Alternatively, you can leave your sequence alignment in
standard NEXUS or PHYLIP format and enter the organism, strain, chromosome, etc.
information on the following
Organism Page
.
>Nucleotide Page
#The options on this page will vary depending on the
Submission Type
and
Sequence Data Format
selected earlier. Gapped sequences must be imported as properly
formatted FASTA files. Details about importing alignment files are
below
.
\*Nucleotide Page for FASTA Data Format
\*\*Create Alignment
#If you have selected a Population study, Phylogenetic study, Mutation
study, or Environmental samples set as a
Submission Type
a check box will appear at the top of the Nucleotide Page. If you
check 'Create Alignment', Sequin will attempt to generate an alignment
of the sequences within your submission. Alignments are not required
for these types of submissions however.
\*\*Import Nucleotide FASTA
#Use this button to import your properly formatted
FASTA file
. You will see a window containing information about the imported
sequence(s). Please check the number of sequences, Sequence IDs
(SeqIDs) and length of each sequence to make sure they are correct. If
you have included source information within the FASTA definition line,
this will also be listed.
\*\*Add/Modify Sequences
#This option allows you to add or modify sequences without using a
previously formatted FASTA file, but is not available if you have
selected a gapped sequence as a
Submission Type
. On the Specify Sequences box you can either import a nucleotide FASTA
or add a new sequence. If you choose Add New Sequence, a new box will
pop-up where you can either import an existing sequence file or
directly paste or type the nucleotide sequence.
#If you add a sequence where the FASTA definition line is not properly
formatted a pop-up box will appear. The top box in this pop-up will
list any errors in the FASTA definition lines, including missing
SeqIDs, duplicate SeqIDs for different sequences, or improperly
formatted modifiers. You can add or edit this information in the
spreadsheet provided. The toggle at the bottom of the pop-up allows
you to select whether all sequences or only those with errors are
listed in the spreadsheet above. After making changes, click on
Refresh Error List to ensure that all errors have been corrected. You
must correct any errors involving the SeqID in order to proceed with
your submission. Click on Accept to save your sequences and return to
the Specify Sequences box.
#In the Specify Sequences box, you can choose to add another sequence or
select a sequence from the list and choose to edit or delete it. You
can also delete all sequences at this point. You will need to click on
Done to save your sequences and return to the Nucleotide Page.
\*\*Clear Sequences
#This option will remove all imported nucleotide sequences.
\*\*Specify Molecule
#A database sequence can represent one of several different molecule
types. The default molecule is genomic DNA. If the sequence was not
derived from genomic DNA, you can edit that information here. If you
are submitting multiple sequences you can apply one molecule type to
all sequences or apply the molecule type to each sequence individually.
Enter in the Molecule pop-up menu the type of molecule that was
sequenced.
#-Genomic DNA: Sequence derived directly from the DNA of an organism.
Note: The DNA sequence of an rRNA gene has this molecule type, as does
that from a naturally-occurring plasmid.
#-Genomic RNA: Sequence derived directly from the genomic RNA of certain
organisms, such as viruses.
#-Precursor RNA: An RNA transcript before it is processed into mRNA,
rRNA, tRNA, or other cellular RNA species.
#-mRNA[cDNA]: A cDNA sequence derived from mRNA.
#-Ribosomal RNA: A sequence derived from the RNA in ribosomes. This
should only be selected if the RNA itself was isolated and sequenced.
If the gene for the ribosomal RNA was sequence, select Genomic DNA.
#-Transfer RNA: A sequence derived from the RNA in a transfer RNA, for
example, the sequence of a cDNA derived from tRNA.
#-Other-Genetic: A synthetically derived sequence including cloning
vectors and tagged fusion constructs.
#-cRNA: A sequence derived from complementary RNA transcribed from DNA,
mainly used for viral submissions.
#-Transcribed RNA: A sequence derived from any transcribed RNA not
listed above.
#-Tranfer-messenger RNA: A sequence derived from transfer-messenger RNA,
which acts as a tRNA first and then an mRNA that encodes a peptide tag.
If the gene for the tmRNA was sequenced, use genomic DNA.
#-ncRNA: A sequence derived from other non-coding RNA not specified
#above. If the gene for the ncRNA was sequenced, select Genomic DNA.
\*\*Specify Topology
#Most sequences have a Linear topology and this is the default. You
should change this setting to Circular only if the sequence is complete
and it has a circular topology. For example, a complete plasmid or a
complete mitochondrial genome would have a Circular topology, but a
single gene from a plasmid or mitochondrion would have a Linear
topology. If you are submitting multiple sequences you can apply one
topology to all sequences or set the topology for each sequence
individually.
\*Nucleotide Page for Aligned Data Formats
\*\*Import Nucleotide Alignment
#Once you have imported the alignment using the Import Nucleotide
Alignment button, you can edit the molecule information using the
Specify Molecule
and
Specify Topology
buttons explained above. Note that you can not access the
Add/Modify Sequences
dialog for submissions of aligned sequences.
\*\*Optional Alignment Settings
#If you are submitting a set of aligned sequences, you can specify sequence
characters used in your alignment here. In most cases, the default
settings do not need to be changed.
#Every sequence within an alignment file must contain the same number of
characters (nucleotides + gaps). Gap characters are used to represent the
spaces between contiguous nucleotides in an alignment. Gaps that appear at
the beginning or end of a sequence are treated differently than gaps that
appear between nucleotides and each must be defined. GenBank prefers to
use a hyphen (-) to represent gaps. If you use a different character to
represent a gap, you will need to add this character to the list in the
Beginning Gap, Middle Gap, or End Gap boxes.
#Ambiguous characters represent nucleotides that are known to exist, but
whose identity has not been experimentally validated. GenBank prefers to
use 'n' to represent any ambiguous nucleotides. If you are using a
different character to represent an ambiguous base, you will need to add
this character to the list in the Ambiguous/Unknown box. Sequin will
convert these characters to 'n's when your file is imported.
#Match characters denote nucleotides that are identical in every member of
an alignment. GenBank prefers the use of a colon (:) to represent match
characters. If you are using a different character to represent a match
character, you will need to add this character to the list in the Match box.
>Organism Page
#Information about the organism from which the sequence was derived
should be entered or edited on this page. If there are any potential
problems with the organism information previously provided in either
the
FASTA definition line
or entered in the
Add/Modify Sequences
dialog, a window listing these problems will appear at the top of the
form. Please review these problems and edit using the

Add Source Modifiers button as necessary. At minimum, you must supply
the scientific name of the organism from which the sequence was
obtained in order to proceed with your submission.
#The second window is a summary of the organism information provided so
far. Double clicking on a line of text within this window will launch a
modifier-specific editing window. In each of these windows, you can
edit the available information for the specific modifier. In most
cases, you have the choice to edit the modifier for each sequence
separately, or to enter text and select Apply above value to all
sequences. These changes will be reflected in the windows of the
Organism page immediately upon closing the modifier-specific editor.
\*Add Organisms, Locations, and Genetic Codes
#If you have not added organism information using either the
FASTA definition line
or the
Add/Modify Sequences
dialog, you can use the Add Organisms, Locations, and Genetic Codes to
do so at this point. This button will launch the Multiple Organism
Editor pop-up where you may add or edit existing information concerning
the
Organism
name,
Location
and
Genetic Code
. The SeqID of each sequence is listed at the left of the spreadsheet
format. You can change the information in the spreadsheet individually
or globally for all sequences.
\*\*Organism
#The scrollable list at the top of the pop-up contains the scientific
names of many organisms. To reach a name on the list, type the first
few letters of the scientific name into the box above the list or the
appropriate box in the spreadsheet. The list will scroll to the names
beginning with those letters, and you can select the organism within
the list itself. You can then use the arrow button to copy this name
into the appropriate box in the spreadsheet.
#To apply the same scientific name to all sequences in the submission,
click on the Organism button in the spreadsheet column header. A
separate pop-up box will appear with the same organism list. You can
select a name from this list and choose Accept to apply this name to
all sequences.
#If you have any questions about the scientific name of an organism, see
the NCBI
Taxonomy Browser
http://www.ncbi.nlm.nih.gov/Taxonomy/tax.html
#If the name of the organism is not on the list, type it in directly. If
you do not know the scientific name, please be as specific as you can
and include a unique identifier, such as a clone, isolate, strain or
voucher number, or cultivar name, e.g.; Nostoc ATCC29106, uncultured
spirochete Im403, Lauraceae sp. V�squez 25230 (MO), Rosa hybrid
cultivar 'Kazanlik'. Also, if applicable, indicate if the name is
unpublished as of the time of submission. Additional information such
as strain, isolate, or serotype can be entered later in the submission
process.
\*\*Location
#The default Location for all sequences is "Genomic". If the sequence
is not genomic, select the alternative location (ie, organelle) from
the pull-down list. You can change the location of all sequences
globally by clicking on the Location button in the spreadsheet header.
The following is a brief description of the choices in this list:
#-Apicoplast: a reduced plastid characteristic of apicomplexans
(e.g., Plasmodium). NOTE: apicoplast should be applied ONLY to
members of the Apicomplexa.
#-Chloroplast: a chlorophyllous plastid.
#-Chromatophore: a membrane-bound vesicle containing photosynthetic pigments
in bacteria.
#-Chromoplast: a non-chlorophyllous, pigmented plastid, found in
fruits and flowers.
#-Cyanelle: a specialized type of plastid found exclusively in
glaucocystophytes (e.g., Cyanophora). NOTE: cyanelle should be
applied ONLY to members of the Glaucocystophyceae.
#-Endogenous\_virus: a virus that has integrated permanently into the
host genome, and which is inherited vertically through the
germline of the host.
#-Extrachromosomal: other extrachromosomal elements not listed here,
such as a B chromosome or an F factor.
#-Genomic: chromosome. This category includes
mitochondrial and chloroplast proteins that are encoded by the nuclear
genome.
#-Hydrogenosome: an organelle that produces hydrogen and ATP and is
found mainly in ciliates, fungi and trichomonads. Hydrogenosomes may
be reduced mitochondria.
#-Kinetoplast: a specialized type of mitochondrion found exclusively
in Kinetoplastida (e.g., Leishmania). NOTE: kinetoplast should
be applied ONLY to members of the Kinetoplastida (trypanosomes and
bodonids).
#-Leucoplast: a plastid lacking pigments of any type.
#-Macronuclear: a specialized type of nucleus found exclusively in the
ciliated protists (e.g., Tetrahymena). NOTE: macronucleus
should be applied ONLY to members of the Ciliophora.
#-Mitochondrion: a semi-autonomous, self-reproducing organelle that
occurs in the cytoplasm of most eukaryotic cells.
#-Nucleomorph: a reduced nuclear remnant found in Chlorarachniophyceae
(e.g., Chlorarachnion) and Cryptophyta (e.g, Cryptomonas). NOTE:
nucleomorph should be applied ONLY to members of the
Chlorarachniophyceae or Cryptophyta.
#-Plasmid: extrachromosomal genetic element found in bacterial species.
Note this does not include the cloning vector used to propagate
the sequence of interest.
#-Plastid: any of a class of double membrane-bound, light-harvesting
organelles (or derived from same). NOTE: plastid should be used
ONLY when a more precise term, e.g., chloroplast, is not
applicable.
#-Proplastid: an immature plastid.
#-Proviral: a virus that is integrated into a host cell chromosome.
\*\*Genetic Code
#If you selected a scientific organism name from the scrollable list
described above, this field will be filled out automatically. However,
if the organism is not on the list, this field will default to the
"Standard" genetic code. If this is incorrect, you can select the
correct genetic code from the pull-down list. To globally change the
genetic code for all sequences which are not automatically filled out,
click on the Genetic Code button in the spreadsheet header.
#For more information regarding the genetic codes available, see the NCBI
Taxonomy page
.
http://www.ncbi.nlm.nih.gov/htbin-post/Taxonomy/wprintgc?mode=c
\*Import Source Modifiers
#Using this button allows you to import a tab-delimited table of source
modifiers. The first column in the table must contain the Sequence
Identifiers (SeqIDs) used earlier in the submission and each subsequent
column must contain a different source modifier. The first row in the
table must contain the labels for each column. The label for the
Sequence Identifiers column should be in the format "Seq\_ID". A list
of
modifiers
in the format to be used in the column headers is available.
\*Add Source Modifiers
#Using this button will launch the Specify Source Modifiers pop-up box
where you can add or edit any source modifier. You can also import a
source modifier table or export the existing source modifiers in table
format from this page.
#The Select Modifier dialog allows you to select a modifier from the
pull-down list and edit the value of this modifier for each sequence or
globally add a value to all sequences.
#The two windows in this pop-up provide information about the current
source modifiers for the sequences in your submission. The top window
provides a summary of these modifiers and the lower window lists the
values of each modifier for each sequence. If any sequences have
missing organism names or have source information that is identical to
another sequence, the SeqIDs will be shown in red in this window.
Double-clicking on a modifier value in this window will launch a pop-up
where you can edit this value. Double-clicking on the modifier name
used in the header will launch a modifier-specific pop-up where you can
globally edit the modifier value for all sequences or change the value
for individual sequences.
\*Clear All Source Modifiers
#This button will clear all modifiers previously entered in either the
FASTA definition lines or the submission dialogs. This includes the
organism name which is required for submission.
>Protein Page
#This page allows you to provide the protein sequence translated from
the nucleotide sequence that you just entered. If the nucleotide
sequence is alternatively spliced or contains multiple open reading
frames, enter all of the protein sequences on this page. Each protein
sequence will appear in the database record as a coding sequence (CDS)
feature. Sequin will automatically determine which nucleotide
sequences code for the protein and indicate the nucleotide sequence
interval on the database record. Sequin also provides tools that allow
you to view a graphical representation of all the open reading frames
in your nucleotide sequence and to convert these reading frames into
CDS features. These tools are described later in the help
documentation under the
ORF Finder.
\*Conceptual Translation Confirmed by Peptide Sequencing
#Most protein entries are computer-generated conceptual translations of
a nucleic acid sequence. If you have confirmed this translation by
direct sequencing either of the entire protein or of peptides derived
from the protein, please check this box.
\*Incomplete at NH3 end/Incomplete at COOH end
#If the sequence is lacking amino acids at the amino- or
carboxy-terminal end of the protein, please check the appropriate box.
\*Create Initial mRNA with CDS Intervals
#If you check this box, Sequin will make an mRNA feature with the same
initial intervals (i.e., range of sequence) as the CDS feature. After
the record has been assembled, you should edit the mRNA feature location
to add the 5' UTR and 3' UTR intervals. This may be done either in the
mRNA editor or in the sequence editor.
\*Import Protein FASTA
#You can import a single or multiple protein sequences contained within
a previously generated protein FASTA file.
\*\*FASTA Format for Protein Sequences
#The basic FASTA format is the same as that used for
nucleotide sequences
, with a FASTA definition line followed by the sequence itself.
#In order to match the protein sequence to the correct nucleotide
sequence, you must use the same Sequence Identifier (SeqID) that you
used to identify the nucleotide sequence. Thus in cases of
alternatively spliced genes, a single protein FASTA file can contain
two unique sequences that have the same SeqID. Both coding regions
will be added to the same nucleotide sequence.
#The available modifiers for use in a protein FASTA definition line are
different than those for a nucleotide FASTA definition line and are
limited to information about the protein or gene itself and are
contained within the examples below. The format remains [modifer=text].
#Note in all cases, the FASTA definition line must not contain any hard
returns. All information must be on a single line of text.
#Examples of properly formatted protein FASTA definition lines are:
 ```` ```
>Seq1 [protein=neuropilin 1] [gene=Nrp1]
``` ```` 
 ```` ```
>ABCD [protein=merozoite surface protein 2] [gene=msp2] [protein_desc=MSP2]
``` ```` 
 ```` ```
>DNA.new [protein=breast and ovarian cancer susceptibility protein] [gene=BRCA1] [note=breast cancer 1, early onset]
``` ```` 
#The protein name should be included in the entry; all other fields are
optional.
#The line after the FASTA definition line begins the amino acid
sequence. It is recommended that each line of sequence be no longer
than 80 characters. Please only use IUPAC symbols within the amino
acid sequence. Non-IUPAC amino acid symbols will be stripped from the
sequence.
#After you import your sequence, a window will appear with information
about the sequence. The first line will describe the number of protein
sequences imported and the total length in amino acids of
all sequences. Each sequence is numbered, and its length,
unique identifier (SeqID), Gene symbol, Protein name, and title
(Definition line) as supplied in the FASTA definition line are listed.
>Annotation Page
#Note: This page will not be available if you have selected a gapped
sequence as the
Submission Type
.
#On this page, you can add a
CDS
,
gene
or
ribosomal RNA
feature across the entire span of each sequence you are submitting.
You can not specify locations within each sequence using this page. In
order to add a CDS feature, you must enter a protein name. More options
are available under the
Annotate Menu
in the record viewer.
#If the feature should be partial at one or both ends, check the
appropriate box and then fill in the text boxes for the relevant
feature. You can also select whether the feature is on the Plus
or Minus strand.
#You may add a title to all sequences if this was not included in the
FASTA definition line. This will be used as the DEFINITION field in
the final flatfile. The title should contain a brief description of
the sequence.
>Assembly Tracking
#You will only see this form if you had previously indicated that the
entry is a Third-Party Annotation submission. You must provide the
GenBank Accession number(s) of the primary sequence used to assemble
your TPA sequence. We can not accept primary sequences corresponding
to Reference Sequences or those from proprietary databases. More
information about this can be found on the
TPA
home page.
#If a proper GenBank Accession is entered in the first column of the
Assembly Tracking form, the GenBank staff can map the coordinates for
you. You do not need to fill out the 'from' and 'to' columns. Note
that multiple accessions may be entered to provide full coverage of the
assembled sequence.
#If the accession entered is not recognized as a GenBank Accession
number, a pop-up box is generated requesting that you edit the numbers
listed. Sequences from the trace archive can be used primary sequence
data for TPA records but must be entered in the format "TI123456789".
#You may also generate an Assembly Tracking form in the record viewer
under the Annotate menu. Select Descriptors and TPA Assembly from the
pull-down menu in order to generate the Assembly Tracking form.
>Editing the Record
\*Overview
#After you finish the Organism and Sequences Form, Sequin will process
your entry based on the information you have entered. The window you
see now is called the record viewer. This is also the window you will
see if you are submitting an update to an existing record. The
instructions after this point are the same whether you are submitting a
new record or an update.
#In the default window of the record viewer, you will see your entry
approximately as it would appear in the database. Most of the
information that you entered earlier in the submission process is
present in the viewer; other information, such as the contact, is still
present in the record but will not be visible in the database entry. If
you have provided a conceptual translation of the nucleotide sequence,
the translation will be listed as a CDS Feature. Sequin automatically
determines which nucleotides encode for the protein, and lists them,
even if the nucleotide sequence contains introns and exons.
#You can save the entry to a file by selecting Save or Save As under the
File menu. This is not the same as saving the entry for submission to
the database. It is a good idea to save the file at this point so that
if you make any unwanted changes during the editing process you can
revert to the original copy. If you wish to edit the entry later, click
on "Read Existing Record" on the Welcome to Sequin form and choose
the file.
#It is likely that the entry could be processed now for submission to
the database. However, you may wish to add information to
the entry. This information may be in the form of Descriptors or
Features. Descriptors are annotations that apply to an
entire sequence, or an entire set of sequences, and Features are
annotations that apply to a specific sequence interval. For example,
you may want to change the Reference Descriptor to add a published
manuscript, or to annotate the sequence by adding features such as a
signal peptide or polyA signal.
#Information in the record viewer can be edited in different ways. One
way to modify information is to double click within the block of
information you wish to edit. Many blocks, such as "Definition",
"Source", "Reference", or "Features" can be edited.
#To add information, create a new descriptor
or feature by selecting the appropriate form from the Misc or Features
menus. These options are described later in this help document.
#Finally, you may need to edit the sequence itself.
Instructions
for working with the sequence are presented in the documentation for the
Sequence Editor.
\*Submitting the Finished Record to the Database
#Once you are satisfied that you have added all the appropriate
information, you must process your entry for submission to the database.
Select "Validate" under the Search menu. This function detects
discrepancies between the format of your submission and that required by
the database selected for entry.
#If Sequin detects problems with the format of your record, you will see a
screen listing the validation errors as well as suggestions for how to fix the
discrepancies. Single clicking on an error message scrolls the record viewer
to the feature that is causing the error. Double clicking on the error
message launches the relevant feature editor on which you can correct the
problem. If you are annotating a set of multiple sequences, shift-click to
scroll to the target sequence and feature. When you think you have corrected
all the problems, click on "Revalidate". You can submit files with errors,
but it is strongly recommended that you correct as many errors as possible
prior to submission.
#Message: Select Verbose, Normal, Terse, or Table. Verbose gives a more
detailed explanation of the problem.
#Filter: Select the error messages you wish to see. You can select
ALL, SEQ\_INST (errors regarding the sequence itself, its type, or
length), SEQ\_DESCR (descriptor errors), SEQ\_FEAT (feature errors), or
errors specific to your record.
#Severity: Select the types of error messages you wish to see. You
will see the type of message selected, as well as any messages warning
of more serious problems.
#There are four types of error messages, Info, Warning, Error, and
Reject. Info is the least severe, and Reject is the most severe. You
may submit the record even if it does contain errors. However, we
encourage you to fix as many problems as possible. Note that some
messages may be merely suggestions, not discrepancies. A possible
Warning message is that a splice site does not match the consensus.
This may be a legitimate result, but you may wish to recheck the
sequence. A possible Error message is that the conceptual translation
of the sequence that you supplied does not encode an open reading
frame. In this case, you should check that you translated the sequence
in the correct reading frame. A possible Reject message is that you
neglected to include the name of the organism from which the sequence
was derived. The name of the organism is absolutely required for a
database entry.
#If Sequin does not detect any problems with the format of your record,
you will see a message that "Validation test succeeded".
#To prepare the submission, click the "Done" button on the record
viewer, or select "Prepare Submission" under the File menu. You will be
prompted to save the file. Email this file to the database at the
address shown. You MUST email the file; Sequin does not submit the
file automatically over the network. The email addresses for the
databases are:
!-GenBank: gb-sub@ncbi.nlm.nih.gov
!-EMBL: datasubs@ebi.ac.uk
#After your entry is complete, close the record viewer. You will be
returned to the Welcome to Sequin form and can begin another entry.
>The Record Viewer
\*Target Sequence
#This pop-up menu shows a list of SeqIDs of all nucleotide and protein
sequences associated with the Sequin entry. Use the menu to select the
sequences displayed in the record viewer, as well as the sequences you
want to "target", that is, the sequences to which you want to apply a
descriptor (see
Descriptors
in the Sequin help documentation). You may select either an individual
sequence by name or a set of sequences, such as All Sequences, or
SEG\_dna if you have a segmented nucleotide set. You may change the
selection at any time.
\*Display Format
#You may change the display format of the record viewer to any of the
formats described below. Editing a field in one display format will
change that field in all formats. Subsequent pop-up menus will appear
depending on which format is selected.
\*\*GenBank
#This display format allows you to see the submission as it would appear
as a GenBank or DDBJ entry. It is the default format.
#The Mode pop-up default setting is Sequin. Release mode shows certain
qualifiers and db\_xrefs in RefSeq entries which are non-collaborative.
Entrez mode is used for web display and can show new elements that have
not yet finished their four month quarentine period. Dump mode requires
that the accession slot be populated. In most cases, there is no need
to change from the default Sequin mode.
#The Style pop-up allows different views of segmented records. The
default is Normal. Segment style is the traditional representation of
segmented sequences, while Contig style displays a CONTIG line with a
join of accessions instead of raw sequence. Master style shows
features mapped to the segmented sequence coordinates instead of the
coordinates of the individual parts.
\*\*Graphic
#This display format shows the entry in a graphical view. The top bar
represents the nucleotide sequence. Lower arrows or bars represent
different features on the sequence. Double click on an arrow or bar to
launch the appropriate editing window. Any sequence highlighted in the
Sequence Editor will be boxed on the graphical view of the sequence.
To see a graphical representation of a segmented set (see
Submission type
,
above), the Target Sequence must be set to
SEG\_dna.
#The Style pop-up menu allows you to see the display in different styles
and colors.
#The Scale pop-up menu allows you to see the display in different sizes.
The smaller the number, the larger the display.
\*\*Sequence
#This display format shows the nucleotide sequence in the record along
with any annotated features (such as CDS or mRNA). You can only view a
single sequence at a time with this option. You can use the Features
pop-up menu to change the display of the features. With the numbering
pop-up menu, select where you want the sequence numbers to be
indicated, at the side of the window, at the top of each sequence line,
or not at all.
\*\*Alignment
#This display format shows sets of aligned sequences, such as those
imported as part of a population, phylogenetic, mutation, or
environmental samples set. When toggled to All Sequences in the Target
Sequence pop-up, the alignment of all entries will be displayed. To
more closely analyze similarities, you can select a single entry in the
Target Sequence pop-up. The complete sequence of the entry selected
will be displayed. Any nucleotides in the other sequences that differ
from that selected will be displayed, while identical nucleotides will
be displayed as a period. You can also display features annotated on
the selected target sequence or all sequences using the Feature display
toggle. To launch the alignment editor, select
Alignment Assistant
from the record viewer Edit menu.
\*\*EMBL
#This display format allows you to see the submission as it would appear
as an EMBL entry.
\*\*Table
#This display format shows the annotation in a five-column, tab-delimited
table
format. This format can be imported to add annotation to a record that
has none.
\*\*FASTA
#This display shows the sequence and Definition line only, without any
annotations, in a format called the FASTA format. This is a format used
by many molecular biology analysis programs. You cannot edit in this
display mode.
\*\*Quality
#This display format shows quality score data ifit has been included in
the submission.
\*\*ASN.1
#This display shows the entry in Abstract Syntax Notation 1, a data
description language used by the NCBI. You cannot edit in this display
mode.
\*\*XML
#This display format shows the entry in XML language, sometimes used by
various databases. You cannot edit in this display mode.
\*\*INSDSeq
#This display format shows the entry in the XML format used by the INSD.
You cannot edit in this display mode.
\*\*Desktop
#The NCBI DeskTop displays the internal
structure of the record being viewed in Sequin. The
DeskTop
is explained under the Misc menu.
\*Done
#This button allows you to validate the entry when you are finished with
the submission. See
Submitting the Finished Record to the Database
in the Sequin help documentation.
\*Controls for Downloaded Entries
#If you have downloaded a sequence from Entrez, you will see an
additional button labeled PubMed. This button will launch a web
browser containing the target sequence as it appears in Entrez. From
here, you can access any Entrez-supported Links, including related
sequences and associated references in PubMed.
>Descriptors
\*Overview
#Descriptors are annotations that apply to an entire sequence, or an
entire set of sequences, in a given entry. They do not have a specific
location on a sequence, as they apply to the entire sequence. They can
be contrasted to
Features,
which apply to a specific interval of the sequence.
#You may edit descriptors in one of two ways.
#(1) In the record viewer, double click within the text of the
descriptor to bring up a form on which information can be added.
#(2) Choose the option Descriptors from the Annotate menu.
\*Annotate Menu - Descriptors
#This menu allows you either to create new descriptors or to modify
existing ones. Select the descriptor that you wish to modify.
#When you first select a descriptor, you will see a window called
"Descriptor Target Control". Using the target control pop-up menu,
select the sequences you wish this descriptor to cover. The name(s)
listed correspond to the SeqID(s) given to the nucleotide or amino acid
sequences when they were imported into Sequin. The default selection
for this menu is set in the Target Sequence pop-up menu on the record
viewer. You may choose to have the descriptor cover just one sequence,
or a set of sequences in your entry. If you are creating a new
descriptor, select "Create New". If you wish to modify a previous
descriptor, select "Edit Old".
#The following is a list of some of the descriptors that can be added.
Two additional descriptors, those for
Publications
and
Biological Source,
are described in other sections.
\*\*TPA Assembly
#If you indicated that your sequence is a TPA submission, a
TPA Assembly
was created from the information regarding primary accession numbers.
This Assembly information can be edited here. Note that it is not
necessary to enter nucleotide location in the "from" and "to" columns.
\*\*Update Date
#This is for database staff use only. Please do not modify the date.
\*\*Create Date
#This is for database staff use only. Please do not modify the date.
\*\*Region
#This descriptor provides general information about the genetic context
of the sequence. For example, if your nucleotide sequence is cloned
from the region surrounding the Huntington's Disease gene, you could
enter that information here. Providing information for this descriptor
is optional.
\*\*Name
#Alternative place for a descriptive name for the sequence. This
information will not appear in the flatfile view, but will be
maintained in the ASN1.
\*\*Comment
#This descriptor is used to list any additional information that you
wish to provide about the sequence. Use of this descriptor is optional.
Most information can be better annotated using the appropriate
features and qualifiers rather than a generic comment descriptor.
\*\*Title
#This descriptor contains the information that will go on the Definition
line of the database entry. If you supplied a title for your nucleotide
sequence when you imported it into Sequin, that information is here. If
you wish to change the Definition line, or if you did not supply a title
when you submitted the sequence, edit this Descriptor. Note that
nucleotide definition lines follow a standard format and may be changed
by the staff when your submission is processed.
\*\*Molecule Description
#This descriptor indicates the characteristics of the molecule from
which the sequence was derived. The information that you have already
entered can be edited here. In most cases, the molecule and class are
the only choices which should be edited from the default values.
\*\*\*Molecule
#A GenBank sequence can represent one of several different molecule
types. Enter in the Molecule pop-up menu the type of molecule that was
sequenced. A brief description of the choices in this pop-up menu were
listed previously.
\*\*\*Completedness
Choose the appropriate option from the pop-up menu.
#-Complete: Use this designation when a complete molecule, such as a
complete mitochondrial genome, is being submitted.
#-Partial: Use this designation when an incomplete unit, such as the
partial coding sequence of a gene, is being submitted.
#-No left: Use this designation when an incomplete unit, such as the
partial coding sequence of a gene, or a partial protein sequence, is
being submitted. The sequence has no left if it is incomplete on the
5', or amino-terminal, end.
#-No right: Use this designation when an incomplete unit, such as the
partial coding sequence of a gene, or a partial protein sequence, is
being submitted. The sequence has no right if it is incomplete on the
3', or carboxy-terminal, end.
#-No ends: Use this designation when an incomplete unit, such as the
partial coding sequence of a gene, or a partial protein sequence, is
being submitted, The sequence has no ends if it is incomplete at both
the 5' and 3', or amino- and carboxy- terminal, ends.
#-Other: Use this designation when none of the above descriptions apply.
\*\*\*Technique
#From the pop-up menu, select the technique that was used to generate the
sequence.
#-Standard: standard sequencing technique.
#-EST:
Expressed Sequence Tag
: single-pass, low-quality mRNA sequences
derived from cDNAs. These sequences will appear in the EST division.
#-STS:
Sequence Tagged Site
: short sequences that are operationally
unique in a genome and that define a specific position on the physical
map. These sequences will appear in the STS division.
#-Survey:
single-pass genomic sequence
. These sequences will appear in
the Genome Survey Sequence (GSS) division.
#-Genetic Map: Genetic map information, for example, in the Genomes division.
#-Physical Map: Physical map information, for example in the Genomes division.
#-Derived: A sequence assembled into a contig from shorter sequences.
#-Concept-trans: A protein translation generated with the appropriate
genetic code.
#-Seq-pept: Protein sequence was generated by direct sequencing of a
peptide.
#-Both: Protein sequence was generated by conceptual translation and
confirmed by peptide sequencing.
#-Seq-pept-Overlap: Protein sequence was generated by sequencing
multiple peptides, and the order of peptides was determined by overlap
in their sequences.
#-Seq-pept-Homol: Protein sequence was generated by sequencing
multiple peptides, and the order of peptides was determined by homology
with another protein.
#-Concept-Trans-A: Conceptual translation of the nucleotide sequence
provided by the author of the entry.
#-HTGS 0:
High Throughput Genome Sequence
, Phase 0. These sequences
are produced by high-throughput sequencing projects and will be in the
HTG division.
#-HTGS 1:
High Throughput Genome Sequence
, Phase 1. These sequences
are produced by high-throughput sequencing projects and will be in the
HTG division.
#-HTGS 2:
High Throughput Genome Sequence
, Phase 2. These sequences
are produced by high-throughput sequencing projects and will be in the
HTG division.
#-HTGS 3:
High Throughput Genome Sequence
, Phase 3. These sequences
are produced by high-throughput sequencing projects and will be in the
HTG division.
#-FLI\_cDNA: Full Length Insert cDNA. Sequence corresponds to entire cDNA but
not necessarily entire transcript. These sequences are produced by large
sequencing projects.
#-HTC: High Throughput cDNA. These sequences are produced by large sequencing
projects.
#-WGS:
Whole Genome Shotgun
. These sequences are produced by large sequencing projets and follow a
separate submission process.
#-Barcode: Nucleotide sequence is part of Barcodes of Life project. This
selection should only be used by members of the Consortium for the
Barcodes of Life.
#-Composite-WGS-HTGS: Nucleotide sequence has been assembled by large
sequencing centers using a combination of whole genome shotgun and BAC-baed
sequencing.
#-TSA: Transcriptome Shotgun Assembly. Shotgun assemblies of mRNA sequences
from primary data submitted to dbEST, the short read archive (SRA) or the
trace archive.
#-Other: Do not use this designation.
\*\*\*Class
#From the pop-up menu, select the type of molecule that was sequenced.
#-DNA: DNA
#-RNA: RNA
#-Protein: Protein
#-Nucleotide: Do not select this item
#-Other: Do not select this item
\*\*\*Topology
#From the pop-up menu, select the topology of the sequenced molecule.
#-Linear: Linear molecule (most sequences).
#-Circular: Circular molecule (such as a complete plasmid or mitochondrion).
#-Tandem: Do not select this item.
#-Other: Do not select this item.
\*\*\*Strand
#From the pop-up menu, select whether the sequence was derived from an
organism with a single- or double-stranded genome. This is used primarily for
viral submissions.
#-Single: The organism contains only a single-stranded genome, for
example, ssRNA viruses.
#-Double: The organism contains only a double-stranded genome, for
example, dsDNA viruses.
#-Mixed: Do not select this item.
#-Mixed Rev: Do not select this item.
#-Other: Do not select this item.
\*\*Biological Source
#The Biological Source descriptor is described in more detail
below.
>Features
\*Overview
#Features are annotations which apply to one or more intervals on a
sequence. They can be contrasted to
Descriptors,
that apply to an entire sequence or an entire set of sequences.
Features will be added to the Target Sequence selected in the record
viewer pop-up menu.
#You may add or modify features in one of three ways.
#(1) In the record viewer, double click on the text of an existing
feature to bring up a form on which information can be added or edited.
#(2) Choose the feature from the Annotate menu to add a new feature.
#(3) Choose the feature from the Sequence Editor Features menu to add a
new feature.
#The features listed in the Annotate menu and the Sequence Editor
Features menu are identical, and the instructions for adding them are
the same, with one exception. If you annotate them in the Annotate
menu, you must provide the nucleotide sequence location of the feature.
However, if you add features from the Sequence Editor, you can
highlight the sequence that the feature covers, and the location of the
sequence will be automatically entered in the feature location box.
\*Annotate Menu - Features
#This menu allows you to add or modify features on the sequence selected
in the Target Sequence pop-up menu of the record viewer. Features are
grouped into six categories. Select the feature that you would like to
mark on your sequence. A new form will appear.
#Feature forms share a common design. The first page is specific to the
particular feature, e.g., Coding Region or Gene. The second page lists
Properties of the Feature. The third page describes the Location of the
feature. Details about the common second and third pages are provided
below.
\*\*Properties Page
\*\*\*General Subpage
#Enter general comments about the feature here.
#Select any of the flags if necessary. If this sequence contains only a
partial representation of the feature you are describing, check the
"Partial" box. Check the "Exception" box if the feature annotates a
post-transcriptional modification of the nucleotide sequence, such as
ribosomal slippage or RNA editing. This is generally used only on CDS
features. The evidence dialogs will only be editable if information
has been entered in the Evidence subpage.
#If a gene feature overlaps the feature you are editing, the gene symbol
will appear in the pull-down menu. If you want to add the name of a
new gene, select new, and enter its name and optional description. By
default, mapping between the feature and the gene is done by overlap,
that is, the gene associated with the feature is the gene whose
location overlaps with the location of the feature. Under some
circumstances, for example, if the sequences of two genes overlap, you
may wish the feature to apply to a different gene. In this case,
select cross-reference, and select the name of the new gene in the
pop-up menu. If you do not want the feature to map to any existing
gene, select suppress. You may also edit information on the Gene
feature form by clicking on Edit Gene Feature.
\*\*\*Comment Subpage
#Add any comments about the feature here, especially if you checked the
"Exception" box on the General Subpage.
\*\*\*Citations Subpage
#This page is used to list any citations that specifically apply to the
feature you are annotating. The citation must have already been entered
into the record (see
Publications
)
in the Sequin help documentation. Click on Edit Citations, and
place a check mark in box next to the publication you want to cite.
However, we discourage the use of citations on features.
\*\*\*Cross-Refs Subpage
#This is a read-only page used to cross-reference this entry to entries
in external databases (databases other than GenBank, EMBL/EBI, and
DDBJ), such as dbEST or FLYBASE. For more information on this topic,
see the International Nucleotide Sequence Database Collaboration
page
.
http://www.ncbi.nlm.nih.gov/collab/db\_xref.html
\*\*\*Evidence Subpage
#This page is primarily used by large sequencing centers to explain
annotation prediction methods and its use is optional. More details
about these qualifiers can be found in the
genome submission guidelines
.
The two choices of evidence are Experiment or Inference.
#Wet-bench, experimental evidence can be entered as free text in the
Experiment section. Please be as brief as possible.
#The Inference section allows for information to be added in cases where
the feature is annotated based solely on sequence similarity or
prediction software. In order to fill in text, you must select one of
the options from the Type pull-down menu. The Category field is
optional and indicates which category of data is inferred. Different
pull-down and text boxes will appear depending on the selection you
choose from the Type menu. If you select one of the 'similar to'
categories, you must include the name of the database and the
corresponding accession number of the sequence used as the basis for the
annotation. If you choose one of the prediction categories, you must
include the name and version of the prediction program used as the basis
for the annotation.
#For example, if your annotation of a coding region was based on
similarity to the sequence and annotation in GenBank Accession number
AY411252, you would select "similar to DNA sequence" from the pull-down
menu and then select "INSD" in the Database pull-down. You would then
type "AY411252.1" in the Accession text box. If the annotation is
based on the Genscan prediction algorithm, you would select "ab initio
prediction" from the pull-down menu, select "Genscan" in the Program
pull-down and enter 2.0 in the Program Version text box. If the
database or program used is not listed in the appropriate pull-down
list, select Other from the list. A new text box will appear where you
can enter the name of the database or program used. You still must
include the appropriate accession number or version in the subsequent
text box.
\*\*\*Identifiers Subpage
#This is a read-only page used by the database staff for tracking
features within the record.
\*\*Location Page
#This page allows you to select the location of the feature you are
citing. Each feature must have a sequence interval associated with it.
In most cases, Sequin will limit the option to the nucleic acid or
protein sequence as appropriate.
#Check the 5' Partial or 3' Partial box if the feature in your nucleic
acid sequence is missing residues at the 5' or 3' ends, respectively.
Check the NH2 Partial or COOH Partial if the feature in your amino acid
sequence is missing residues at the amino- or carboxy-terminal ends,
respectively. If you checked "Partial" on the Properties page, you
must check either the 5' and/or 3' partial boxes.
#Enter the sequence range of the feature. The numbers should correspond
to the nucleotide sequence interval if the SeqID is set to a nucleotide
sequence, and to an amino acid sequence interval if the SeqID is set to
a protein sequence. If the feature spans multiple, non-continuous
intervals on the sequence, indicate the beginning and end points of each
interval. If each interval is separate, and should not be joined with
the others to describe the feature, check the Intersperse intervals with
gaps box (for example, when annotating multiple primer binding sites).
If the feature is composed of several intervals that should all be
joined together, do not check the box (for example, when annotating mRNA
on a genomic DNA sequence).
#For nucleic acid Features only: From the pop-up menu, select the
strand on which the feature is found.
#-Plus: Plus strand, or coding strand.
#-Minus: Minus strand, or non-coding strand.
#-Both: Both strands.
#-Reverse: Do not select this item.
#-Other: Do not select this item.
#Use the pop-up menu to select the SeqID of the sequence you are
describing by the location. Clicking on the X button to the left will clear
location spans, strand, and SeqID from that row.
#If you are working on a set of sequences which contain an alignment,
you will see a toggle at the bottom of the Location Page where you can
select to add or view the location of the feature using the Sequence
Coordinates of the target sequence or the Alignment Coordinates. In
either case, the feature will only be added to the target sequence. If
you want to add features to all members of the set using the alignment
coordinates, you must use the
Alignment Assistant
.
#A brief description of the available features follows. A detailed
explanation of how to use the coding region (CDS) feature is included.
The DDBJ/EMBL/GenBank feature table definition
page
http://www.ncbi.nlm.nih.gov/collab/FT/index.html
provides detailed information about other features.
\*attenuator
#1) region of DNA at which regulation of termination of transcription
occurs, which controls the expression of some bacterial operons; 2)
sequence segment located between the promoter and the first structural
gene that causes partial termination of transcription.
\*C\_region
#Constant region of immunoglobulin light and heavy chains, and T-cell
receptor alpha, beta, and gamma chains. Includes one or more exons,
depending on the particular chain.
\*CAAT\_signal
#CAAT box; part of a conserved sequence located about 75 bp upstream of
the start point of eukaryotic transcription units that may be involved
in RNA polymerase binding; consensus=GG(C or T)CAATCT.
\*CDS
#coding sequence; sequence of nucleotides that corresponds with the
sequence of amino acids in a protein (location includes stop codon).
Feature includes amino acid conceptual translation.
\*\*Coding Region Page
#Most users add a coding region to their sequence when they fill out the
Organism and Sequences form. However, you may need to edit the coding
region, or add additional ones. Choose CDS under the Coding Regions
and Transcripts submenu of the Features menu, or to edit an existing
CDS, double click on the record viewer. If you appended the partial
sequence of a coding region to the Organism and Sequences form, you will
probably need to edit the Coding Region feature to avoid validation
error messages about the location of the coding region.
\*\*\*General (Product) Subpage
#Choose the genetic code that should be used to translate the
nucleotide sequence. For more information, and for the translation
tables themselves, see the NCBI Taxonomy
page
.
If the genetic code is already populated from the taxonomy database, do
not change this selection.
#Choose the reading frame in which to translate the sequence. Do not
fill in the Protein Product or SeqID selections.
#Supply additional information about the protein by clicking on Edit
Protein Information to launch the Protein feature forms. The protein
name must have already been filled out on the Protein subpage.
#If you have changed the nucleotide location spans of the coding region,
check Update mRNA Span on Accept. This will adjust the mRNA feature
locations as well.
#Checking retranslate on accept will translate the nucleotide sequence
according to the interval(s) indicated on the Locations page when you
click on Accept to exit the editor. This new translation will replace
any earlier translations you have supplied. This should not be a
problem if the interval was indicated appropriately.
#If the coding sequence that you supply is a partial sequence and you
have checked a Partial box on the Location subpage, it is a good idea to
check the Synchronize Partials box. In this case, Sequin will ensure
that all other appropriate features (such as protein) are also marked as
partial.
#When editing existing CDS features, choose the sequence you want to
view by selecting its name uder the Product pop-up menu. You may also
import a new protein sequence by selecting Import Protein FASTA under
the file menu. The sequence should be formatted as described above on
the Organism and Sequences form.
#After you have imported a protein sequence, click on Predict Interval.
This function will predict the interval on the nucleotide sequence to
which the coding region applies. If you do not select this function,
the interval will likely be wrong, and you will get an error message
when you attempt to validate the record. If your sequence is a 5' or 3'
partial, you must first indicate this manually on the Location Page.
#You may also have Sequin generate the protein sequence from the
nucleotide sequence by clicking on Translate Product. However, you must
first indicate the location and partialness of the coding region on the
Location page in order to obtain the correct translation.
#The Edit Protein Sequence button will launch an amino acid
Sequence Editor
as discussed below.
#The Adjust for Stop Codon button will truncate a displayed translation
at the first stop codon. If no stop codon is present in the current
translation, this function will extend the translation to the first stop
codon or to the end of the sequence. In both cases, the spans of the
coding region will be automatically updated on the Location Page to
reflect the new translation.
\*\*\*Protein Subpage
#Use this page to enter or edit a name or description of the protein
product. For a new sequence, enter information directly into the
boxes. You can edit descriptions of an existing sequence by clicking
on Edit Protein Feature which will bring up the Protein feature form.
The Launch Product Viewer displays the flatfile view of ht eprotein
record generated from the information in the CDS feature.
\*\*\*Exceptions Subpage
#Exceptions describe places where there is a posttranslational
modification. Enter the amino acid position at which the modification
occurs, and select the amino acid that is actually represented in the
protein from the pop-up list. Sequin will change the amino acid number
to a nucleotide interval. Please provide some explanation for the
exception in a comment.
\*D-loop
#Displacement loop; a region within mitochondrial DNA in which a short
stretch of RNA is paired with one strand of DNA, displacing the
original partner DNA strand in this region; also used to describe the
displacement of a region of one strand of duplex DNA by a single
stranded invader in the reaction catalyzed by RecA protein.
\*D\_segment
#Diversity segment of immunoglobulin heavy chain, and T-cell receptor
beta chain.
\*enhancer
#A cis-acting sequence that increases the utilization of (some)
eukaryotic promoters and can function in either orientation and in any
location (upstream or downstream) relative to the promoter.
\*exon
#Region of genome that codes for portion of spliced mRNA; may contain
5' UTR, all CDSs, and 3' UTR.
\*gap
#Gap in the sequence, only applied to gaps of unknown length. The
location span of the gap feature is 100 base pairs, indicated by 100 "n"s
in the sequence. The qualifier /estimated\_length=unknown is mandatory.
\*GC\_signal
#GC box; a conserved GC-rich region located upstream of the start point
of eukaryotic transcription units that may occur in multiple copies or
in either orientation; consensus=GGGCGG.
\*gene
#Region of biological interest identified as a gene and for which a name
has been assigned.
\*iDNA
#Intervening DNA; DNA which is eliminated through any of several kinds
of recombination.
\*intron
#A segment of DNA that is transcribed, but removed from within the
transcript, by splicing together the sequences (exons) on either side of
it.
\*J\_segment
#Joining segment of immunoglobulin light and heavy chains, and T-cell
receptor alpha, beta, and gamma chains.
\*LTR
#Long terminal repeat, a sequence directly repeated at both ends of a
defined sequence, of the sort typically found in retroviruses.
\*mat\_peptide
#Mature peptide or protein coding sequence; coding sequence for the
mature or final peptide or protein product following post-translational
modification. The location does not include the stop codon (unlike the
corresponding CDS).
\*misc\_binding
#Site in nucleic acid that covalently or non-covalently binds another
moiety that cannot be described by any other Binding key (primer\_bind or
protein\_bind).
\*misc\_difference
#Feature sequence is different from that presented in the entry and
cannot be described by any other Difference key (unsure, mutation,
variation, or modified\_base).
\*misc\_feature
#Region of biological interest which cannot be described by any other
feature key.
\*misc\_recomb
#Site of any generalized, site-specific, or replicative recombination
event where there is a breakage and reunion of duplex DNA that cannot be
described by other recombination keys (iDNA and virion) or qualifiers of
source key (/proviral).
\*misc\_RNA
#Any transcript or RNA product that cannot be defined by other RNA keys
(prim\_transcript, precursor\_RNA, mRNA, 5'UTR, 3'UTR,
exon, transit\_peptide, polyA\_site, rRNA, tRNA, and ncRNA).
\*misc\_signal
#Any region containing a signal controlling or altering gene function or
expression that cannot be described by other Signal keys (promoter,
CAAT\_signal, TATA\_signal, -35\_signal, -10\_signal, GC\_signal, RBS,
polyA\_signal, enhancer, attenuator, terminator, and rep\_origin).
\*misc\_structure
#Any secondary or tertiary structure or conformation that cannot be
described by other Structure keys (stem\_loop and D-loop).
\*mobile\_element
#Region of genome containing an element capable of or derived from
movement from one location to another in the genome. The
mobile\_element\_type qualifier is mandatory and a pull-down menu lists
approved types. The name of the specific element can be given in the
text box.
\*modified\_base
#The indicated nucleotide is a modified nucleotide and should be
substituted for by the indicated molecule (given in the mod\_base
qualifier value).
\*mRNA
#messenger RNA; includes 5' untranslated region (5' UTR), coding sequences
(CDS, exon) and 3' untranslated region (3' UTR).
\*ncRNA
#non-coding RNA; a non-protein-coding transcript other than ribosomal RNA and
transfer RNA, including antisense RNA, guide RNA, scRNA, siRNA, miRNA, piRNA,
snoRNA, and snRNA. The specific type of ncRNA must be specified in the
/ncRNA\_class qualifier.
\*N\_region
#Extra nucleotides inserted between rearranged immunoglobulin segments.
\*operon
#Region containing polycistronic transcript under the control of the same
regulatory sequences.
\*oriT
Origin of transfer; region of DNA where transfer is initiated during the
process of conjugation or mobilization.
\*polyA\_signal
#Recognition region necessary for endonuclease cleavage of an RNA
transcript that is followed by polyadenylation; consensus=AATAAA.
\*polyA\_site
#Site on an RNA transcript to which will be added adenine residues by
post-transcriptional polyadenylation.
\*precursor\_RNA
#Any RNA species that is not yet the mature RNA product; may include 5'
clipped region (5' clip), 5' untranslated region (5' UTR), coding
sequences (CDS, exon), intervening sequences (intron), 3' untranslated
region (3' UTR), and 3' clipped region (3' clip).
\*prim\_transcript
#Primary (initial, unprocessed) transcript; includes 5' clipped region
(5' clip), 5' untranslated region (5' UTR), coding sequences (CDS, exon),
intervening sequences (intron), 3' untranslated region (3' UTR), and 3'
clipped region (3' clip).
\*primer\_bind
#Non-covalent primer binding site for initiation of replication,
transcription, or reverse transcription. Includes site(s) for synthetic
e.g., PCR primer elements.
\*promoter
#Region on a DNA molecule involved in RNA polymerase binding to initiate
transcription.
\*protein\_bind
#Non-covalent protein binding site on nucleic acid.
\*RBS
#Ribosome binding site.
\*repeat\_region
#Region of genome containing repeating units. Some qualifiers such as
rpt\_type and satellite have controlled vocabularies. These
qualifiers have check boxes or pull-down menus to ensure that the
correct format is used.
\*rep\_origin
#Origin of replication; starting site for duplication of nucleic acid to
give two identical copies.
\*rRNA
#Mature ribosomal RNA ; the RNA component of the ribonucleoprotein
particle (ribosome) that assembles amino acids into proteins.
\*S\_region
#Switch region of immunoglobulin heavy chains. Involved in the
rearrangement of heavy chain DNA leading to the expression of a
different immunoglobulin class from the same B-cell.
\*sig\_peptide
#Signal peptide coding sequence; coding sequence for an N-terminal
domain of a secreted protein; this domain is involved in attaching
nascent polypeptide to the membrane; leader sequence.
\*source
#Identifies the biological source of the specified span of the sequence.
This key is mandatory. Every entry will have, as a minimum, a single
source key spanning the entire sequence. More than one source key per
sequence is permittable.
\*stem\_loop
#Hairpin; a double-helical region formed by base-pairing between
adjacent (inverted) complementary sequences in a single strand of RNA or
DNA.
\*STS
#Sequence Tagged Site. Short, single-copy DNA sequence that
characterizes a mapping landmark on the genome and can be detected by
PCR. A region of the genome can be mapped by determining the order of a
series of STSs.
\*TATA\_signal
#TATA box; Goldberg-Hogness box; a conserved AT-rich septamer found
about 25 bp before the start point of each eukaryotic RNA polymerase II
transcript unit that may be involved in positioning the enzyme for
correct initiation; consensus=TATA(A or T)A(A or T).
\*terminator
#Sequence of DNA located either at the end of the transcript or adjacent
to a promoter region that causes RNA polymerase to terminate
transcription; may also be site of binding of repressor protein.
\*tmRNA
#Transfer messenger RNA; acts as a tRNA first, then an mRNA that encodes a
peptide tag.
\*transit\_peptide
#Transit peptide coding sequence; coding sequence for an N-terminal
domain of a nuclear-encoded organellar protein; this domain is involved
in post- translational import of the protein into the organelle.
\*tRNA
#Mature transfer RNA, a small RNA molecule (75-85 bases long) that
mediates the translation of a nucleic acid sequence into an amino acid
sequence.
\*unsure
#Author is unsure of exact sequence in this region.
\*V\_region
#Variable region of immunoglobulin light and heavy chains, and T-cell
receptor alpha, beta, and gamma chains. Codes for the variable amino
terminal portion. Can be made up from V\_segments, D\_segments,
N\_regions, and J\_segments.
\*V\_segment
#Variable segment of immunoglobulin light and heavy chains, and T-cell
receptor alpha, beta, and gamma chains. Codes for most of the variable
region (V\_region) and the last few amino acids of the leader peptide.
\*variation
#A related strain contains stable mutations from the same gene (e.g.,
RFLPs, polymorphisms, etc.) that differ from the presented sequence at
this location (and possibly others).
\*3'UTR
#Region near or at the 3' end of a mature transcript (usually following
the stop codon) that is not translated into a protein; trailer.
\*5'UTR
#Region near or at the 5' end of a mature transcript (usually preceding
the initiation codon) that is not translated into a protein; leader.
\* -10\_signal
#Pribnow box; a conserved region about 10 bp upstream of the start point
of bacterial transcription units that may be involved in binding RNA
polymerase; consensus=TAtAaT.
\* -35\_signal
#A conserved hexamer about 35 bp upstream of the start point of
bacterial transcription units; consensus = TTGACa or TGTTGACA.
>Biological Source Descriptor or Feature
#This annotation is very important, as an entry cannot be processed by
the databases unless it includes some basic information about the
organism from which the sequence was derived. This basic information was
entered previously in the submission, in the Organism and Sequences
Form. The more detailed Organism Information form allows you to alter
or add to the data you entered earlier.
\*Overview: Descriptor or Feature?
#Sequin allows two types of biological source information to be entered,
Biological Source Descriptors and Biological Source Features. Biological
Source Descriptors, like other descriptors, provide organism information
about an entire sequence, or an entire set of sequences, in an entry.
Biological Source Features, like other features, provide organism
information about a specific interval on a given sequence.
#In most cases, you will want to use a Biological Source Descriptor, because
all the sequences in the entry will derive from the same source. However, if
you have sequenced a transgenic molecule, for example, one that is part plant
and part bacterial, you would use Biological Source Features to annotate which
sequence was derived from plant and which from bacteria.
#To add a Biological Source Descriptor, select Biological Source under
the Descriptor section of the Annotate menu. To add a Biological
Source Feature, select Biological Source under the Bibliographic and
Comments section of the Annotate menu.
#Annotating a Biological Source Descriptor or Feature is similar to
annotating any descriptor or feature. For help in creating descriptors
and features, see the appropriate section of the help documentation.
The following are instructions for filling out Biological
Source-specific forms.
\*Organism Page
\*\*Names Subpage
#The scrollable list contains the scientific names of many organisms.
To reach a name on the list, either type the first few letters of the
scientific name, or use the thumb bar. Click on a name from the list to
fill out the scientific name field. If there is a common name for the
organism, that field will be filled out automatically. You may also
directly type in the scientific name. If you have any questions about
the scientific or common name of an organism, see the NCBI
taxonomy browser
http://www.ncbi.nlm.nih.gov/Taxonomy/tax.html
\*\*Location Subpage
\*\*\*Location of Sequence
#From the selection list, please enter the location of the genome that
contains your sequence. Most entries will have a "Genomic" location.
A brief description of the choices in this pop-up menu were listed
previously.
\*\*\*Origin of Sequence
#This menu is for the use of database personnel. Please leave this
field empty. The Biological focus box should be checked in rare cases
where multiple source features are annotated.
\*\*Genetic Codes Subpage
#Please use these fields to select the nuclear and mitochondrial genetic
code that should be used to translate the nucleic acid sequence. The
genetic code for a eukaryotic organism is "Standard". If you selected
an organism name from the scrollable list described above, this field
was filled out automatically. Do not change these fields if they have
been filled out automatically.
#For more information regarding the translation tables available, see
the NCBI Taxonomy
page
.
\*\*Lineage Subpage
#This information is normally entered by the database staff. They will
use the
Taxonomy database
http://www.ncbi.nlm.nih.gov/Taxonomy/tax.html
maintained by the NCBI/GenBank.
#If you disagree with the lineage supplied please notify the database
staff.
#If you are running Sequin in its
network-aware
mode, you will see a button labeled "Lookup Taxonomy". Click on this
button to perform an automatic look-up of the taxonomic lineage of the
organism. Sequin will perform the look-up by accessing the Taxonomy
database and will fill out the Taxonomic Lineage and
Division fields.
#If you have any comments about the taxonomic lineage determined by
Sequin, please submit these comments with your entry. Under the Sequin
File menu, select Edit Submitter Info. Enter your comments in the box
entitled "Special Instructions to Database Staff", on the Submission
page.
\*Modifiers Page
#This page allows you to enter additional information about the source
and/or organism. Entering information is optional.
\*\*Source Subpage
#Choose a modifier from the pull-down menu on the left side of the page
and type the appropriate name on the right side of the page. If you do
not find appropriate modifiers in the scroll down list, you can enter
additional source information as text in the field at the bottom of the
page. You may add multiple modifiers to describe the source organism.
#Clicking on the X button to the right of the text box will remove the
text and clear the modifier from the pull-down in that line.
#If the sequence was determined from a PCR product, check the PCR primers
box at the bottom of the page. This will launch a separate editor where
you can enter the direction, name and sequence of all PCR primers. The
Set field allows you to group which primers were used in the same PCR
reaction. All primers used in the same reaction should list the same
number under Set. This dialog is for PCR primers only, not for
sequencing or other primers.
#The following is a description of the available modifiers:
#-Cell-line: Cell line from which sequence derives.
#-Cell-type: Type of cell from which sequence derives.
#-Chromosome: Chromosome to which the gene maps.
#-Clone: Name of clone from which sequence was obtained.
#-Clone-lib: Name of library from which sequence was obtained.
#-Collected-by: Name of person who collected sample. Do not use
accented or non-ASCII characters.
#-Collection-date: Date sample was collected. Must use format
23-Mar-2005, Mar-2005, or 2005.
#-Country: The country of origin of DNA samples used for epidemiological
or population studies. A list of approved country designations can
be found on the
ISDC web pages. Additional text may be added after a colon. For example,
/country="USA: Bethesda, MD"
#-Dev-stage: Developmental stage of organism.
#-Endogenous-virus-name: Name of inactive virus that is integrated into
the chromosome of its host cell and can therefore exhibit vertical
transmission.
#-Environmental-sample: Identifies sequence derived by direct molecular
isolation from an unidentified organism. You cannot include extra text when
using this modifier; the text box will change to TRUE upon selection of this
modifier from the pull-down list
#-Frequency: Frequency of occurrence of a feature.
#-Genotype: Genotype of the organism.
#-Germline: If the sequence shown is DNA and a member of the
immunoglobulin family, this qualifier is used to denote that the sequence
is from unrearranged DNA. You cannot include extra text when using this
modifier; the text box will change to TRUE upon selection of this modifier
from the pull-down list.
#-Haplogroup: Combination of stable polymorphic variants clustered together
in a specific combination which can indicate a common ancestor.
#-Haplotype: Haplotype of the organism.
#-Identified-by: Name of person who identified sample. Do not use
accented or non-ASCII characters.
#-Isolation-source: Describes the local geographical source of the organism
from which the sequence was derived
#-Lab-host: Laboratory host used to propagate the organism from which
the sequence was derived.
#-Lat-Lon: Latitude and longitude of location where sample was
collected. Mandatory format is decimal degrees N/S E/W. Selecting this
modifier in the pull-down list will generate separate boxes for entering the
information in the mandatory format.
#-Linkage-group: Group of genes whose loci are physically connected and tend
to segregate together during meiosis.
#-Map: Map location of the gene.
#-Mating-type: Designation of individual single-celled organisms and protists
based on mating behavior.
#-Metagenomic: Identifies sequence from a culture-independent genomic
analysis of an environmental sample submitted as part of a whole genome
shotgun project. You may not include extra text when using this modifier,
instead the text box will change to TRUE upon selection.
#-Plasmid-name: Name of plasmid from which the sequence was obtained.
#-Pop-variant: Name of the population variant from which the sequence was
obtained.
#-Rearranged: If the sequence shown is DNA and a member of the
immunoglobulin family, this qualifier is used to denote that the sequence
is from rearranged DNA. You cannot include extra text when using this
modifier; the text box will change to TRUE upon selection of this modifier
from the pull-down list.
#-Segment: Name of viral genome fragmented into two or more nucleic acid
molecules.
#-Sex: Sex of the organism from which the sequence derives.
#-Subclone: Name of subclone from which sequence was obtained.
#-Tissue-lib: Tissue library from which the sequence was obtained.
#-Tissue-type: Type of tissue from which sequence derives.
#-Transgenic: Identifies organism was the recipient of transgenic
DNA. You cannot include extra text when using this modifier; the text box
will change to TRUE upon selection of this modifier from the pull-down list.
\*\*Organism Subpage
#Choose a modifier from the pull-down menu on the left side of the page
and type the appropriate name on the right side of the page. If you do
not find appropriate modifiers in the scroll down list, you can enter
additional organism information as text in the field at the bottom of
the page. You may add multiple modifiers to describe the source organism.
#Clicking on the X button to the right of the text box will remove the text
and clear the modifier from the pull-down in that line.
#The following is a description of the available modifiers:
#-Acronym: Standard synonym (usually of a virus) based on the initials
of the formal name. An example is HIV-1.
#-Anamorph: The scientific name applied to the asexual phase of a fungus.
#-Authority: The author or authors of the organism name from which sequence
was obtained.
#-Bio-material: An identifier of the stored biological material from which
the sequence was obtained. This qualifier should be used to cite collections
that are not appropriate in specimen-voucher or culture-collection. Examples
include stock centers and seed banks. Mandatory format is "institution
code:collection code:material\_id". However, only material\_id is required.
Selecting this modifier in the pull-down list will generate separate boxes for
entering the information in the correct format.
#-Biotype: See biovar.
#-Biovar: Variety of a species (usually a fungus, bacteria, or virus)
characterized by some specific biological property (often geographical,
ecological, or physiological). Same as biotype.
#-Breed: The named breed from which sequence was obtained (usually applied
to domesticated mammals).
#-Chemovar: Variety of a species (usually a fungus, bacteria, or virus)
characterized by its biochemical properties.
#-Common: Common name of the organism from which sequence was obtained.
#-Cultivar: Cultivated variety of plant from which sequence was obtained.
#-Culture-collection: Identifier and institution code of the microbial or
viral culture or stored cell-line from which the sequence was obtained. This
qualifier should be used to cite the collection where the author has deposited
the culture or from which the culture was obtained. Personal library
collections should be annotated in strain and not in culture-collection.
Mandatory format is "institution code:collection code:culture\_id". However,
collection code is not required. Selecting this modifier in the pull-down
list will generate separate boxes for entering the information in the correct
format.
#-Ecotype: The named ecotype (population adapted to a local habitat) from
which sequence was obtained (customarily applied to populations of
Arabidopsis thaliana).
#-Forma: The forma (lowest taxonomic unit governed by the nomenclatural
codes) of organism from which sequence was obtained. This term is usually
applied to plants and fungi.
#-Forma-specialis: The physiologically distinct form from which sequence
was obtained (usually restricted to certain parasitic fungi).
#-Group: Do not select this item.
#-Host: Natural (as opposed to laboratory) host to the organism from which
sequenced molecule was obtained. Use of the Latin name of the host organism
is preferred.
#-Isolate: Identification or description of the specific individual
from which this sequence was obtained. An example is Patient X14.
#-Metagenome-source: Used only for genome projects. Do not select this item.
#-Pathovar: Variety of a species (usually a fungus, bacteria or virus)
characterized by the biological target of the pathogen. Examples
include Pseudomonas syringae pathovar tomato and Pseudomonas syringae
pathovar tabaci.
#-Serogroup: See serotype.
#-Serotype: Variety of a species (usually a fungus, bacteria, or virus)
characterized by its antigenic properties. Same as serogroup and
serovar.
#-Serovar: See serotype.
#-Specimen-voucher: Identifier of the physical specimen from which the
sequence was obtained. The qualifier is intended for use where the sample is
still available in a curated museum, herbarium, frozen tissue collection, or
personal collection. Mandatory format is "institution code:collection
code:specimen\_id". However, only specimen\_id is required. Selecting this
modifier in the pull-down list will generate separate boxes for entering the
information in the correct format.
#-Strain: Strain of organism from which sequence was obtained.
#-Subgroup: Do not select this item.
#-Sub-species: Subspecies of organism from which sequence was obtained.
#-Substrain: Sub-strain of organism from which sequence was obtained.
#-Subtype: Subtype of organism from which sequence was obtained.
#-Synonym: The synonym (alternate scientific name) of the organism name
from which sequence was obtained.
#-Teleomorph: The scientific name applied to the sexual phase of a fungus.
#-Type: Type of organism from which sequence was obtained.
#-Variety: Variety of organism from which sequence was obtained.
\*\*GenBank Subpage
#Please do not use this form. This field is reserved for information from
NCBI's taxonomy database.
\*Miscellaneous Page
\*\*Synonyms Subpage
#If there are alternative names for the organism from which the sequence
was derived, enter them here. Please be aware that this is the
appropriate field only for alternative names for the organism, not for
alternative gene or protein names.
\*\*Cross-Refs Subpage
#This page is for use by database staff only.
>Publications
\*Overview: Descriptor or Feature?
#Sequin allows two types of publications to be entered, Publication
Descriptors and Publication Features. Publication Descriptors are
bibliographic references that, like other descriptors, cover an entire
sequence, or an entire set of sequences, in an entry. Publication
Features are bibliographic references that, like other features, cover
a specific interval on a given sequence.
#Publications are entered into the Reference field of the database
entry. References are citations of unpublished, in press, or published
works that are relevant to the submitted sequence. Publications
should provide information regarding the principle cloning and
determination of the sequence within the record.
#In general, there is one publication describing a sequence, and a
Publication Descriptor should be used. To enter a Publication
Descriptor, select Publications under the Annotate menu and click on
Publication Descriptor.
#However, if one publication describes the cloning of the 5' end of a
gene, and another publication describes the cloning of the 3' end of
the gene, Publication features may be used. To make a publication
feature, choose Publication Feature in the Publications section of the
Annotate menu. Enter the information about the publication, and then
enter the nucleotide interval to which the publication refers on the
Location page.
\*Citation on Entry Form
\*\*Status
#Using the radio buttons, select one of the three options:
#-Unpublished: Select this option if a manuscript has been written but
not yet submitted or has been submitted for publication but has not yet
been accepted.
#-In Press: The article has been accepted for publication but is not yet
in print.
#-Published: The article has been published.
\*\*Class
#Using the radio buttons, select the type of publication in which the
sequence will appear.
#-Journal
#-Book Chapter
#-Book
#-Thesis/Monograph
#-Proceedings Chapter: Abstract from a meeting
#-Proceedings: A meeting
#-Patent
#-Online Publication: Used for journals which publish strictly online and
do not issue print copies.
#-Submission
\*\*Scope
#Using the radio buttons, select one of the options.
#-Refers to the entire sequence: Most publications should be classified
as such.
#-Refers to part of the sequence: For use only when a publication
discusses only part of the presented sequence. You must enter the
locations in the location tab in later forms. This selection is only
valid when adding a Publication feature, not descriptor.
#-Cites a feature on the sequence: This selection should only be made in
limited cases. Its use must coincide with the use of the /citation
qualifier on the given feature.
#After you have filled out the Citation on Entry form, click on
"Proceed" to see the next form.
\*Citation Information Form (General)
\*\*Authors Page
\*\*\*Names Subpage
#Please enter the names of the authors. Note that the first name of the
author is listed first. You can add as many authors to this page as
necessary. After you type in the name of the third author, the box
becomes a spreadsheet, and you can scroll down to the next line by
using the thumb bar. The suffix toggle allows the addition of common
suffixes to the author name. The consortium field should be used when
a consortium is responsible for the sequencing or publication of the
data. The consortium should not be the department or institute
affiliation of the authors. Individual authors may be listed along
with a consortium name.
\*\*\*Affiliation Subpage
#Please enter information about the institution where the sequencing was
performed.
#Other pages in the Citation Information Form will be different,
depending on the Class of publication selected in the Citation on Entry
Form. Instructions for filling out the Citation Information Form for
Journals is included here.
\*Citation Information Form (If Selected Class Was Journal)
\*\*Title Page
#Enter title for manuscript in the box.
\*\*Journal Page
#Fill in the appropriate Journal, Volume, Issue, Pages, Day, and Year
fields by typing information into the boxes. Select the month with the
pop-up menu. If necessary, choose an option from the Erratum pop-up
menu and explain the erratum.
#If you are running Sequin in its
network-aware
mode, the program will look up the Title, Author, and Journal
information in the MEDLINE database if you supply it with some minimal
information. For example, if you know the MUID (MEDLINE Unique
Identifier) of the publication, enter it in the appropriate box and
select "Lookup By MUID." Sequin will automatically retrieve the rest
of the information. One way to find the MUID of the publication is to
look up the publication with the NCBI's
Entrez
service. Alternatively, if you do not know the MUID, enter the Journal,
Volume, Pages, and Year. Then select "Lookup Article". Sequin will
retrieve the missing Title and Author information.
#The PubStatus toggle is used by database staff. If you have used the
"Lookup by MUID" or "Lookup by PMID" functions, this field may be
populated. Please do not edit the information.
\*\*Remark Page
#This page is reserved for use by the database staff.
>File Menu
\*About Sequin
#Details about the current version of Sequin.
\*Help
#Launches the help documentation.
\*Open
#Open an existing entry. This option will open a record that has been
previously saved in Sequin. Furthermore, for analysis purposes, it can also
open
a FASTA-formatted sequence file. The sequence will be displayed in Sequin and
can be analyzed with tools such as CDD Search, but it should not be submitted,
because it does not have the appropriate annotations.
\*Close
#Close this entry.
\*Export GenBank
#Exports the currently displayed format to a file. Do not use export
ASN1 for submission of sequences to the database.
\*Duplicate View
#Duplicates the entry. You can then view the entry simultaneously in
different Display Formats.
\*Save
#Saves the entry. Note: This merely saves the entry so you can go back
and edit it. It does not prepare the entry for submission to the
database, that is, it does not validate the entry.
\*Save As
#See Save.
\*Save as Binary Seq-entry
#Saves the file in a compressed format and should be used only when the
file is to be imported into other analysis programs. Do not use this
option to save files for submission directly to GenBank.
\*Restore
#Replaces the displayed record with a previously saved version. This
feature is useful if you have made unwanted changes since you last saved
the record.
\*Prepare Submission
#Prepares the entry for submission to the database. See
Submitting the Finished Record to the Database
in the Sequin help documentation.
\*Print
#Prints the window that is currently selected. The selected window can
be one of the Sequin forms or pages, or the help documentation.
\*Quit
#Exit from Sequin.
>Edit Menu
\*Copy
#Copy the selected item.
\*Clear
#Clear the selected item.
\*Edit Sequence
#To edit a single sequence, select the sequence identifier in the Target
Sequence pop-up menu, and click on Edit sequence. The sequence editor
will be launched for that sequence. The
sequence editor
is discussed in more detail below.
\*Alignment Assistant
#This option will launch the Alignment Assistant which is discussed in
more detail
below
.
\*Edit Submitter Info
#Opens up the Submission Instructions form, which allows you to enter
additional information about the person submitting the record. Much of
this information was entered on the first form in Sequin, the Submitting
Authors form.
#You can also save the information from the Submitting Authors form
here, so that you can use it in subsequent Sequin submissions. Click
on "Edit Submitter Info" and, under the file menu in the resulting
Submission Instructions form, click on Export Submitter Info to save
the information to a file. For subsequent Sequin submissions, if you
have already saved the submittor information, click on Import Submitter
Info under the File menu on the Submission page of the Submitting
Authors form.
\*\*Submission Page
#Indicate the type of submission. If it is a new submission, select
New. If you are updating an existing submission in order to resubmit it
to the databases, select Update. Check either the "Yes" or "No" radio
button to indicate if the record should be released before publication.
If you select "Yes", the entry will be released to the public after the
database staff has added it to the database. If you select "No", fields
will appear in which you can indicate the date on which the sequences
should be released to the public. The submission will then be held back
until formal publication of the sequence or
GenBank Accession number, or until the Release Date, whichever comes
first. If you have any special instructions, enter them in the box at
the bottom of the page.
\*\*Contact Page
#Update the name, affiliation, or contact numbers of the person
submitting the record. Please supply a fax number to facilitate
communication with database staff.
\*\*Citation Page
#Update the names and affiliation of the people who should receive
scientific credit for the generation of sequences in this entry. The
address should list the principal institution in which the sequencing
and/or analysis was carried out. If you are submitting the record as
an update to the databases, explain the reason for the update on the
Description subpage.
\*Update Sequence
#This selection allows you to replace a sequence with another sequence,
merge two sequences that overlap at their ends, 'patch' a corrected
fragment of a sequence to the current sequence, or copy features from
one sequence to another.
#Use Single Sequence to import a sequence in FASTA or ASN.1 format (for
example, a sequence record that has already been saved in Sequin). If
you are running Sequin in
Network Aware mode,
you can use Download Accession to import a record from Entrez. The
Multiple Sequences option allows you to update multiple sequences using
either FASTA or ASN.1 formats. In either format, each sequence
identifier must be identical in the new and old sequences.
#After you import the updated sequence, a new window will open that
displays two graphical views and the text of the alignment of the new
and old sequence. The first graphic displays the relative length of the
two sequences and the length of the overlapping region between
sequences. The second graphic represents any inserts, deletions, or
point changes within the aligned region between the new and old
sequences. Clicking on a region in this graphic will scroll to the
corresponding nucleotide sequence in the alignment text below.
#The Sequence Update box to the left shows the action that will be
performed upon updating the sequence, i.e., no change, replace, extend
5', extend 3', or patch. The patch function allows you to replace an
internal fragment of the sequence without affecting flanking regions.
You can also override the alignment between the new and old sequence
using the Ignore alignment checkbox to force a sequence change of
replace, Extend 5' or Extend 3'. This option allows you to append new
sequence to with no overlap.
#If the current sequence has annotation, you can use the Existing
Features box to determine whether the annotation should remain or be
removed upon updating the sequence. The Do not remove option is the
default. However, you may chose to remove annotated features only in
the aligned area, outside the aligned area, or to remove all currently
annotated features.
#When updating via Download Accession or an ASN.1 file, the Import
Features box allows you to specify whether features from the new file
should be imported to the existing record. The dialog offers
different options for cases where the features on the new file are
identical to those on the existing record.
#If you are using the Multiple Sequences option, you may choose to
review the sequences and update them one by one using the Update this
Sequence box at the bottom of the window. You may skip a sequence
update or choose to update all sequences at once without reviewing them
in the Update Sequence dialog.
#In any case, please carefully review the sequence and annotation in the
record viewer after using the Update Sequence function.
\*Extend Sequence
#This selection functions similar to the
Update Sequence
function. However, you can only extend the existing sequence in either
the 5' or 3' direction in cases with no overlap between the existing
and new sequences.
\*Feature Propagate
#This selection allows you to propagate any annotated feature from one
sequence in an aligned set to other sequences within the set. For
example, if one nucleotide sequence in the alignment contains a CDS
feature, you can annotate a similar CDS on the other nucleotide
sequences in the set.
#The default source of features to be propagated is the first member
of the set. If you would like to use a different entry as the source of
the features, scope to that entry in the Target Sequence menu before
selecting Feature Propagate from the Edit menu.
#The Feature Propagate window allows you to select which sequences
should receive the new annotation and which features will be
propagated. You can also select whether the features will be extended
or split at gaps in the alignment. The split at gaps selection will
produce two features, one on either side of the gap within the
alignment. If you are propagating a CDS feature, you may specify that
the translation end or extend through internal stop codons. You may
also extend the translation after the stop codon on the source entry by
chosing to translate the CDS after partial 3' boundary. If the CDS
that you are propagating to other records is partial on either end, you
should select the 'Cleanup CDS partials after propagation' check box.
This will retain the partial nature of the CDS features on all records.
The fuse adjacent propagated intervals function will create one
feature from two of the same type that contain abutting nucleotide
intervals due to the nature of the alignment used for propagation.
\*Add Sequence
#This selection allows you to add a new sequence to an existing
population, mutation, phylogenetic, or environmental sample set.
You may import the new entry in FASTA format or ASN.1 format (for
example, a sequence record that has been saved in Sequin).
\*Parse File to Source
#This selection allows you to add unique information for one source
qualifier for each of the records in a batch or set. The input file
must be in the format of a tab-delimited, two column table. The first
column should list the SeqID exactly as it was listed in the original
FASTA file. The second column should list the text value for the
desired source qualifier for each record. Once the file has been
imported, a pop-up box will appear with the source qualifiers listed in
the pull down menus. The qualifiers are separated into three menus:
one for taxonomic information, one for the Organism modifiers and one
for the Source modifiers. For example, in order to add the clone
designations 57 and 49 to the sequences labeled seq1 and seq2, the table
seq1 57
seq2 49
should be used and clone should be selected from the Source modifiers
pull-down menu.
>Search Menu
\*Find ASN.1
#Under this command, you can find and replace strings of letters in
those fields of your submission that contain manually entered data.
The fields that can be altered are Locus, Definition, Accession,
Keywords, Source, Reference, and Features. To use this option, select
Find and fill the Find and Replace lines with the appropriate text.
Note that you cannot edit the sequence in this way.
\*Find FlatFile
#Under this command, you can find strings of letters in all fields of
your submission. You can use the Find First and Find Next buttons to
identify the specified text sequentially through the flatfile.
\*Find by Gene
#This option allows you to move quickly in the record viewer to a gene
feature containing the specified gene symbol.
\*Find by Protein
#This option allows you to move quickly in the record viewer to a CDS
feature containing the specified product name.
\*Find by Position
#This option allows you to move quickly in the record viewer to any
feature annotated at the specified nucleotide location.
\*Validate
#This option detects discrepancies between the format of your submission
and that required by the database selected for entry. If discrepancies
are present, it suggests ways in which to correct them. See the topic on
Submitting the Finished Record to the Database
in the Sequin help documentation.
\*CDD Search
#Performs a CDD BLAST search of the selected sequence against the
NCBI's
Conserved Domain Database
. To do a CDD BLAST search, Sequin must be in its network aware mode.
#CDD currently contains domains derived from two popular collections,
Smart and Pfam, plus contributions from colleagues at NCBI. The source
databases also provide descriptions and links to citations. Since
conserved domains correspond to compact structural units, CDs contain
links to 3D-structure via Cn3D whenever possible.
#The results of the CDD search will be displayed in the record
viewer. These results are for your use only and should be removed
from the record before submission.
\*Vector Screen
#This option allows you to run a BLAST search of your nucleotide
sequence(s) against NCBI's
UniVec
database. We highly recommend that you run this analysis and remove
any vector contamination before submission. The UniVec database
contains only one copy of every unique sequence segment from a large
number of vectors. It also contains sequences for adapters, linkers
and primers commonly used.
#To run Vector screen on a submission containing multiple sequences,
scope to ALL SEQUENCES in the Target Sequence pull-down before running
the analysis. If there are many sequences, a status bar will appear
indicating the progress of the search. If no contamination is found, a
pop-up box will appear to notify you. If contamination is found, a
miscellaneous feature will be annotated on the flatfile with the
location of the contamination. Details will include the relative
strength of the BLAST hit. You must trim the nucleotide sequence to
remove this feature before submission.
\*ORF Finder
#The ORF Finder shows a graphical representation of all open reading
frames (ORFs) in the nucleotide sequence. This tool allows you to
select ORFs and have them appear as coding sequence (CDS) features on
the sequence record.
#The ORFs, indicated by colored boxes, are defined as the longest sequence
containing a start codon and stop codon. All six reading frames are shown as
separate lines in the graphical view. The top three lines represent the plus
strands, and the bottom three lines the minus strands. In the default view,
the nucleotide sequence intervals of the ORFs are displayed in descending
length order on the right side of the window. Intervals on the complementary
(minus) strand are indicated by a 'c'. Selecting 'Order by Start' will
reorder the list based on the nucleotide location of the start codon.
#Clicking on the box labelled ORF changes the graphical display so that the
potential start codons are indicated in white, and stop codons in red.
#The default settings display only those ORFs which contain an ATG start
codon. Selecting 'Alternative' in the 'Initiation Codon' box, will also
include ORFs beginning with all valid alternative start codons as determined
by the genetic code listed in the source feature. If the genetic code for the
source organism has not been specified, the default
standard genetic code
will be used.
#The ORF length button sets the length of ORFs that are
displayed. For example, the default of 10 shows all ORFs that are
greater than 10 nucleotides in length.
#Checking the Show Partial ORFs box will display ORFs that extend to the
end(s) of the nucleotide sequence but are 5' or 3' partial.
#ORFs can be selected by clicking on the graphical representation or on the
sequence interval. Once an ORF has been selected, its location and amino acid
sequence will automatically be fielded in the
CDS feature editor
accessed under the Annotate menu.
\*Select Target
#This option changes the sequence that is selected in the Target
Sequence pop-up. Type the SeqID of the sequence in the box, and the
record viewer will be updated to display that sequence.
>Misc Menu
\*Net Configure
#As a default, Sequin is available as a stand-alone program. However,
the program can also be configured to exchange information with the NCBI
(GenBank) over the Internet. The network-aware mode of Sequin is
identical to the stand-alone mode, but it contains some additional
useful options.
#Sequin will only function in its network-aware mode if the computer on
which it resides has a direct Internet connection. Electronic mail
access to the Internet is insufficient. In general, if you can install
and use a WWW browser on your system, you should be able to install and
use network-aware Sequin. Check with your system administrator or
Internet provider if you are uncertain as to whether you have direct
Internet connectivity.
#There are two ways to change Sequin into its network-aware mode. If
you are still on the initial Welcome to Sequin form, select Net
Configure under the Misc menu. If you have already worked on a Sequin
submission and are looking at the record in the record viewer, select
the Net Configure option from the Misc menu.
#Most users will be able to use the default (Normal) settings on the
Network Configuration page; select Accept to complete the configuration
process.
#If a "Normal" Connection does not work, you may need to select the
Firewall Connection. Contact your system administrator to determine
what to enter into the Proxy and Port fields. If you do not have
access to the domain name server (DNS), uncheck this box.
#The Timeout pop-up selects the length of time that your local copy of
Sequin will wait for a reply from the NCBI server. You may need to set
this number higher (i.e., 60 seconds or 5 minutes) if you are outside
of the United States or have a bad internet connection.
#If you have problems setting up the network configuration, contact
info@ncbi.nlm.nih.gov.
#If you would like to change Sequin back to its stand-alone mode, select
Net Configure again from the Misc menu. Click on Connection: None.
#The network-aware mode of Sequin allows you to perform a number of
additional, important functions. These functions all appear as
additional menu items. A brief description of these functions follows.
Further descriptions are available as indicated elsewhere in the help
documentation.
\*\*Updating Existing GenBank Records
#Using Sequin in its network-aware mode, you can download an existing
GenBank record from Entrez using the GenBank accession number or GI
identification number (NID). You can then use Sequin to make any
necessary changes to the record, and resubmit it to GenBank as a
sequence update.
Instructions
for submitting sequence updates are presented under the Welcome to
Sequin Form. You can download any record from Entrez and look at it in
Sequin. However, you can only formally update those records which you
have submitted since submitters retain editorial control of their
records.
\*\*Performing a PubMed Look-Up
#In its network-aware mode, Sequin can import the relevant sections of a
PubMed record directly into a sequence submission record. Rather than
typing in the entire citation, you can enter minimal information, such
as the PubMed Unique Identifier (PMID), or Journal name, volume, year,
and pages. The
PubMed lookup
is explained in the section of the documentation entitled Publications.
\*\*Performing a Taxonomy Look-up
#In its network-aware mode, Sequin can look
up the taxonomic lineage of an organism from the NCBI's Taxonomy
database. This look-up is normally performed by the NCBI database staff
after the record has been submitted to GenBank. If you look up the
taxonomy before submitting the sequence, you can make a note in the
record of any disagreements. The
taxonomy lookup
is explained in the section of the documentation covering
Biological Source: Organism page: Lineage subpage.
\*\*Accessing the NCBI DeskTop
#The NCBI DeskTop displays the internal
structure of the record being viewed in Sequin. The
DeskTop
is explained under the Misc menu.
\*NCBI DeskTop
#This option is only available if you are running Sequin in its
network-aware
mode.
#The NCBI DeskTop provides a view of the internal structure of the
Sequin record, the ASN.1. Its display resembles a Venn diagram and
represents all the structures represented in the ASN.1 data model.
#In addition, a number of undocumented software tools from the NCBI can
be accessed from the DeskTop. These tools are components of the NCBI
portable software Toolkit. You can also customize these functions using
the Toolkit with your own software tools. The Toolkit and its
documentation are available from the NCBI by anonymous
FTP.
#The DeskTop should only be used by very seasoned users. At this time,
we are not providing any documentation for these specialized functions.
>Annotate Menu
#This menu allows you to enter features and descriptors on the sequence.
#The first six options, Genes and Named Regions, Coding Regions and
Transcripts, Structural RNAs, Bibliographic and Comments, Sites and
Bonds, and Remaining Features refer to types of Features that can be
added to the sequence. Features are described in more detail in the
above section entitled
Features.
#If you are submitting a set of similar sequences, you can add the same
feature across the entire span of each by using the Batch Feature Apply
option. The feature must span the entire nucleotide sequence of each
member; you can not annotate specific nucleotide locations using this
option (for this, see
Feature Propagate).
For each feature, you will be prompted to designate whether the feature
is 5' or 3' partial and whether is is on the plus or minus strand. You
may also add a comment or other qualifier to the feature. The Add
Qualifier option allows you to add a qualifier to an existing feature.
You must specify the feature and qualifier in the Add Qualifier pop-up
box. Source qualifiers can be added to all entries using the Add
Source Qualifier option. Qualifiers specific to the CDS and gene can
be added using Add CDS-Gene-Prot-mRNA and RNA qualifiers using Add RNA
Qual. In each case, a pop-up box appears with qualifier options
appropriate for that feature.
#The Batch Feature Edit function allows you to edit existing qualifiers.
For each menu choice, a pop-up box allows you to select the feature
containing the qualifier and the specific qualifier to be edited. You
can use the Find/Replace text boxes to edit the information contained
within the qualifier.
#Batch Apply Molecule Type allows you to apply the same molecule type to
all nucleotide sequences within your submission. The choices in this
dialog are explained in the
Specify Molecule section of this document.
#The Set Release Date dialog allows you to change the release date
specified in the Submission Dialogs.
#The ORF Finder function is available in both the Annotate and
Search menus.
#Import Source Table allows you to add unique information for one source
qualifier for each of the records in a batch or set. See
Parse File to Source for details.
#The Publications option allows you to add a Publication Feature or
Publication Descriptor to the record. Publications are described in
more detail in the above section entitled
Publications.
#The Descriptors option allows you to add Descriptors to the
record. Descriptors are described in more detail in the section
entitled
Descriptors,
above.
#The Advanced Table Readers option imports a properly formatted
structured comment table. Please contact us if you wish to use this
option.
#Sort Unique Count by Group opens a new window which displays your record(s)
the number of times an individual line appears in the flatfile(s). This is
particularly useful when checking that all records in a large set contain the
required source or feature information.
>Options Menu
#This menu is only available when using Sequin in its network-aware mode.
\*Font
#Use this item to change the display font. From the pop-up menus,
choose the style and size of type. For additional changes, mark the
Bold, Italic, or Underline check boxes. The default font is 10-point
Courier.
>Sequence Editor
#This editor allows you to modify the nucleotide or amino acid sequences
and corresponding annotation in your entry. Although the Sequence Editor
does allow you to undo changes you make to the sequence, we strongly
suggest that you save a copy of the entry before launching the Sequence
Editor so that you can revert to it if necessary.
\*Starting the Sequence Editor
#The sequence that appears in the editor is dependent on the sequence(s)
selected in the Target Sequence pull-down list. There are two ways to
launch the sequence editor for nucleotide sequences. First, you can
double click within sequence in any display format of the record viewer.
A window containing the DNA sequence will appear. Second, in the record
viewer, select the sequence that you would like to edit in the Target
Sequence pop-up menu. Click on Edit Sequence under the Edit menu. You
can launch the editor for protein sequences by selecting the protein
sequence in the Target Sequence pop-up menu and double clicking within
the protein sequence. A window containing the protein sequence will
appear.
\*Moving around the Sequence Editor
#The cursor can be moved with the mouse or the arrow keys. The display
window will change to show the position of the cursor. The sequence
location of the first residue on each line is indicated on the left side
of the window. The cursor location, or the range of sequences selected
by the mouse, is shown in the upper left corner of the window. If you
want to move the cursor to a specific location, type the number in the
box on the top left of the sequence editor window, and hit the Go to
button. If you want to look at a specific sequence, but not move the
cursor to it, type the number in the upper right box of the window and
hit the Look at button.
\*Editing Sequence and Existing Annotation
#Select a piece of sequence by highlighting it with the mouse. To
select the entire sequence, click on a sequence location number on the
left side of the window. Any sequence that is highlighted in the
Sequence Editor will show up as a box on the sequence when it is viewed
in the Graphic Display Format.
#One way to insert and delete residues is with the mouse. Move the
cursor to the appropriate location and type. Text will be inserted to
the left of the cursor. Delete sequence with the backspace or delete
key. Text will be deleted to the left of the cursor. To delete a block
of sequence, highlight it with the mouse and use the delete or backspace
key.
#Another way to insert and delete residues is with options under the Edit
menu of the Sequence Editor. Use Cut to remove, or Copy to copy,
highlighted residues. Copied residues can then be pasted elsewhere
within the sequence by using the Paste option.
#Features annotated via the record viewer will be displayed in a
graphical format within the sequence editor. CDS features will be be
displayed as a blue line across the appropriate nucleotide location. All
other features will be displayed as a black line. To the left of the
line, the name of the feature is displayed. In the case of CDS or mRNA
features, the product name is shown. For gene features, the gene locus
is shown.
#Double-clicking on the feature will launch the feature editor just as in
the record viewer. However, you can also change the nucleotide location
of any feature within the graphical view. To move the entire feature,
select the feature and drag it to the appropriate location while holding
down the mouse button. To alter the 5' or 3' end of a feature, click on
the feature's end and drag to the new location while holding down the
mouse button.
#Before moving the nucleotide locations of a CDS feature, it may be
useful to view the codons in the current translation. You can do this by
clicking on the feature line and releasing the mouse button. A grid will
be displayed that shows the triplet location for the current annotation.
Once you have changed the nucleotide location of a CDS feature in the
graphical view, you can see the new translation by using the Translate
CDS button at the bottom of the window.
#To save changes you have made to the sequence, press the Accept button
at the bottom of the Sequence Editor display window. If you do not want
to save the changes, press the Cancel button at the bottom of the
Sequence Editor display window. Selecting either Accept or Cancel will
quit the Sequence Editor and return you to the record viewer. Any
changes you make will not become a permanent part of the Sequin record
until you Save the record in the record viewer.
#New features can be added using the Features menu.
\*Sequence Editor Window Buttons
\*\*Go to
#Moves the cursor to the indicated location.
\*\*Look at
#Moves the window to the indicated location without moving the cursor.
\*\*Merge Feature Mode/Split Feature Mode
#In merge mode, any new sequence that is entered into a region spanned
by an existing feature becomes part of that feature. For example, if
you enter new sequence in the middle of a CDS, that sequence will be
translated as part of the CDS. In split mode, the new sequence
interrupts the feature. For example, if you enter new sequence in the
middle of a CDS, the CDS will be interrupted by that sequence (see the
location of the CDS in the record viewer).
\*\*Numbering
#Allows the sequence location numbering to be hidden, displayed on the
side, or displayed on the top of the sequence.
\*\*Grid
#Allows the display to show a grid separating each feature and sequence
for easier viewing.
\*\*Show/Hide Features
#This box toggles between hiding and showing the features on a sequence.
\*\*Accept
#Closes the Sequence Editor after saving all of the changes made to
sequences and features.
\*\*Cancel
#Closes the Sequence Editor without saving any changes made to sequences or
features.
\*\*Translate CDS
#Allows translation of coding region features after the location has been
changed within the graphical view.
\*Sequence Editor File Menu
\*\*Export
#Allows the export of a range of sequence as a FASTA file or text file.
Using the text option will also export overlapping features if they are
displayed. If the features are first hidden, only the sequence will be
exported. All protein translations displayed at the time of export, will
be exported as well.
\*\*Accept
#Closes the Sequence Editor after saving all of the changes made to
sequence and features.
\*\*Cancel
#Closes the Sequence Editor without saving any changes made to sequences
of features.
\*Sequence Editor Edit Menu
\*\*Undo
#Undoes all actions performed in the Sequence Editor since the last save.
\*\*Redo
#Restores changes removed with Undo option
\*\*Cut
#Removes the highlighted sequence. This sequence can be pasted elsewhere.
\*\*Paste
#Pastes a cut or copied sequence to the right of the cursor.
\*\*Copy
#Copies the highlighted sequence. This sequence can be pasted elsewhere.
\*\*Find
#Allows you to find DNA or amino acid sequence patterns in your sequence.
The search is case insensitive. To find an exact match to a DNA
sequence pattern, type the pattern in the box. The number of items found
will be displayed and you can toggle through each instance with the Find
Next button. To find the reverse complement of the pattern, click on
the reverse complement box at the bottom of the pop-up box.
#To find an exact match to an amino acid sequence pattern, type that
sequence in the box, and click on "translate sequence". Sequin will look
for all occurrences of that pattern in all six open reading frames. The
DNA sequence encoding that protein sequence in any of the six reading
frames will be hightlighted.
\*\*Translate CDS
#Allows translation of coding region features after the location has been
changed within the graphical view.
\*\*Complement
#Shows the complement of the submitted strand underneath the original.
\*\*Reading Frames
#Shows the indicated phase translation of the selected coding sequence.
You can select any or all of the six reading frames, all reading frames
or all positive or negative frames.
\*\*Protein Mismatches
#Indicates amino acid which does not match conceptual translation
following a nucleotide sequence change. The original amino acid sequence
will be displayed until the Translate CDS function is used. Differences
will be indicated by a red box around the amino acid abbreviation.
\*\*On-the-fly Protein Translations
#Creates a second amino acid sequence in the display which retranslates
as the nucleotide sequence is changed to allow side-by-side comparison to
the original amino acid sequence.
\*Sequence Editor Features Menu
#The menu contains a long list of all features that can be annotated on a
sequence. These features are the same as those that are accessible
through the main Sequin Annotate menu.
#You can annotate features either in the Annotate menu or in the Sequence
Editor. If you annotate them in the Annotate menu, you must type in the
nucleotide sequence location of the feature. However, if you add
features from the Sequence Editor, you can highlight the sequence that
the feature covers, and the location of the sequence will be
automatically entered in the feature location box. Additional
explanations of how to annotate features are provided in the section on
Features.
>Working with Sets of Aligned Sequences
#Sequin allows you to work with aligned sets of closely related
nucleotide sequences that are part of a population, phylogenetic, or
mutation study. If the sequences are imported in a pre-aligned format,
such as PHYLIP, Sequin uses this alignment. If the sequences are
imported individually in FASTA format, Sequin can generate its own
alignment.
#You can view the aligned sequences in the Sequence Alignment Editor. In
the record viewer, select All Sequences in the Target Sequences menu,
and select the Alignment Display Format.
#The Alignment Assistant is launched by selecting Alignment Assistant
from the Edit menu in the record viewer. It can be used to apply
features to the whole set of sequences using the alignment coordinates.
Rather than calculating the nucleotide coordinates for every feature on
every nucleotide sequence, you may select the feature's location using
its alignment coordinates and apply it to every member of the set
simultaneously. Sequin will calculate the nucleotide locations as they
apply to each member of the set.
\*Alignment Assistant Window Buttons
\*\*Go to
#The Go to alignment position and Go to sequence position buttons both
scroll the aligment assistant so that the requested position is
visible. If the requested position is already visible, nothing will
happen. Unlike the Sequence editor window, the 'go to' button does not
control the cursor position.
\*\*Numbering
#Allows the sequence location numbering to be hidden, displayed on the
side, or displayed on the top of the sequence.
\*\*Grid
#Allows the display to show a grid separating each feature and sequence for
easier viewing.
\*\*Features Toggle
#It is possible to view annotated features in the aligment assistant.
The features are displayed as a bar underneath the coordinates for that
feature. The identity of the feature is displayed in the left-hand
column. The default selection is to have the features Hidden. You may
display the features associated only with the Target Sequence or
features annotated on All Sequences in the alignment.
\*Alignment Assistant File Menu
\*\*Export
#Allows you to export the alignment to a file in three different
formats. The contiguous and interleaved options export the alignment
accordingly in FASTA+GAP format. The text representation option saves
the alignment as it appears in the Alignment Assistant. Note that with
this option features are included if they are displayed at the time of
export.
\*\*Close
#Closes the Alignment Assistant window and saves any changes made.
\*Alignment Assistant Edit Menu
\*\*Remove Sequences from Alignment
#Allows you to remove selected sequence(s) from the alignment. Select
the sequence by clicking on it. You can select multiple sequences by
holding down the control key. The sequence will then be highlighted in
grey. Note that this option will remove the sequence from the
alignment, but it is still present in your submission.
\*\*Validate Alignment
#Checks for problems with the alignment. If errors are reported, please
review and attempt to fix your alignment before submission.
\*\*Propagate Features
#This function is the same as that available under the Edit Menu in the
record viewer. A full description is available
above
.
\*Alignment Assistant View Menu
\*\*Target
#Allows you to select a sequence within the alignment as the target
sequence. This can also be done by double-clicking on the sequence
within the alignment. The SeqID of the target sequence will be
displayed in red. Features can be displayed on the target sequence
only and it is the sequence used for comparison in the
Show Substitutions
view.
\*\*Show Substitutions
#Changes the alignment view so that identities are replaced with a "."
and only substitutions are shown. The substitutions and identities are
relative to the selected target sequence.
\*Alignment Assistant Features Menu
#Allows the annotation of features to a single sequence or all sequences
within the alignment. All features available in this menu are
discussed through the main Sequin Annotate menu.
#Select the feature location by clicking the start location on one of
the sequences, keeping the mouse button depressed, drag the cursor to
the end of the feature location. The selected area will now be
underlined and red and the alignment coordinates of this area will be
displayed in the upper left of the Alignment Assistant window.
\*\*Apply to Target Sequence
#Allows you to choose a feature to be applied only to the target
sequence. The locations may be entered manually or can be determined
based on highlighting the sequence as described above.
\*\*Apply to Alignment
#Allows you to add the selected feature to all sequences within your
alignment based on the alignment coordinates you have selected. Note
that in the feature pop-up boxes in this menu, the Location will always
be entered as the location relative to the alignment coordinates.

---


**Questions or Comments?**
  
Write to the NCBI Service
Desk

Revised September 6, 2011
